# Targeting multiple response regulators of *Mycobacterium tuberculosis* augments the host immune response to infection

**DOI:** 10.1038/srep25851

**Published:** 2016-05-16

**Authors:** Srijon Kaushik Banerjee, Manish Kumar, Reshma Alokam, Arun Kumar Sharma, Ayan Chatterjee, Ranjeet Kumar, Sanjaya Kumar Sahu, Kuladip Jana, Ramandeep Singh, Perumal Yogeeswari, Dharmarajan Sriram, Joyoti Basu, Manikuntala Kundu

**Affiliations:** 1 Department of Chemistry, Bose Institute, 93/1 Acharya Prafulla Chandra Road, Kolkata 700009, India; 2 Department of Pharmacy, Birla Institute of Technology & Science-Pilani, Hyderabad Campus, Jawahar Nagar, Hyderabad 500078, India; 3 Division of Molecular Medicine, Bose Institute, P-1/12 CIT Scheme VII M, Kolkata 700054, India; 4 Vaccine and Infectious Disease Research Centre, Translational Health Science and Technology Institute, NCR-Biotech Science Cluster, 3rd Milestone, Faridabad Gurgaon Expressway. Faridabad-121001, India

## Abstract

The genome of *M. tuberculosis* (Mtb) encodes eleven paired two component systems (TCSs) consisting of a sensor kinase (SK) and a response regulator (RR). The SKs sense environmental signals triggering RR-dependent gene expression pathways that enable the bacterium to adapt in the host milieu. We demonstrate that a conserved motif present in the C-terminal domain regulates the DNA binding functions of the OmpR family of Mtb RRs. Molecular docking studies against this motif helped to identify two molecules with a thiazolidine scaffold capable of targeting multiple RRs, and modulating their regulons to attenuate bacterial replication in macrophages. The changes in the bacterial transcriptome extended to an altered immune response with increased autophagy and NO production, leading to compromised survival of Mtb in macrophages. Our findings underscore the promise of targeting multiple RRs as a novel yet unexplored approach for development of new anti-mycobacterial agents particularly against drug-resistant Mtb.

Tuberculosis (TB) caused by *Mycobacterium tuberculosis* (Mtb) continues to represent a major global health problem. According to the World Health Organization, in 2013, 9 million people fell ill with TB, 1.5 million died from the disease and an estimated 4,80,000 people developed multidrug resistant TB^1^. The problem is exacerbated due to HIV-TB co-existence, failure of the BCG vaccine to combat TB and emergence of various drug resistant strains (MDR- and XDR-TB). Novel molecules for chemotherapy are therefore urgently required. The search for new molecules can be driven forward either by screening libraries for their ability to inhibit *in vitro* or intracellular growth of Mtb; or by rational design of molecules directed against targets which are central to the ability of the bacterium to successfully establish infection in the host. The latter approach provides the rationale for engaging in the studies described in this report.

Long term survival of Mtb depends on its ability to sense and adapt to adverse conditions in the host^2^,^3^,^4^,^5^,^6^. Adaptation to environmental signals is associated with transcriptomic changes driven by various regulators including the two-component systems (TCSs)^7^,^8^. The paired TCSs have a sensor histidine kinase (SK) coupled to a response regulator (RR). The sensing of a signal by the SK leads to its autophosphorylation on a histidine residue. Subsequent transfer of the phosphate to an aspartate residue of the cognate RR facilitates binding of the RR to its specific DNA. Each phosphorylated RR regulates a specific repertoire of genes enabling the bacterium to sense and survive under stress. Mtb encodes 11 paired TCSs and a number of orphan RRs and SKs^9^. The TCSs, PhoPR, SenX3/RegX3, PrrAB, and MprAB of Mtb, regulate virulence^9^,^10^. The RRs of these TCSs belong to the OmpR family. The members of this winged helix-turn-helix family of RRs share conserved amino acid residues and structure in the DNA recognition helix^11^,^12^. The PhoP regulon includes genes involved in the synthesis of complex cell wall lipids^13^. SenX3-RegX3 is expressed during phosphate starvation and is required for phosphate uptake and aerobic respiration^14^. PrrAB is required early during intracellular infection^15^. MprAB responds to envelope stress and regulates stress-responsive and virulence-associated genes^16^,^17^. MtrAB is the only essential TCS known so far. It regulates DNA replication and cell division^18^,^19^. We hypothesized that owing to similarities in domain structure and catalytic features, families of these RRs could be targeted by a single molecule resulting in a downstream effect extending across multiple processes such as persistence, reactivation and tuning of host immune responses. Simultaneous disabling of multiple RRs would in turn, compromise bacterial replication and survival.

Using biochemical assays as well as chemical and computational tools we demonstrate that three selected RRs of Mtb, namely MtrA, RegX3 and MprA, belonging to the OmpR family share a common DNA-binding motif and can be targeted by a single molecule, thereby, leading to downstream effects on their regulons, impairment of the type VII ESX-1 secretion machinery, and attenuation of the ability of Mtb to replicate and survive in macrophages. We bring into context how this molecule influences bacterial fate in macrophages by demonstrating that it augments Mtb-induced autophagy and the release of the effector nitric oxide. Its effects on Mtb translate into changes in the immune response to infection.

## Results

### Mutating a common motif in MtrA, MprA and RegX3 abrogates their DNA binding activity

**Figure 1:**
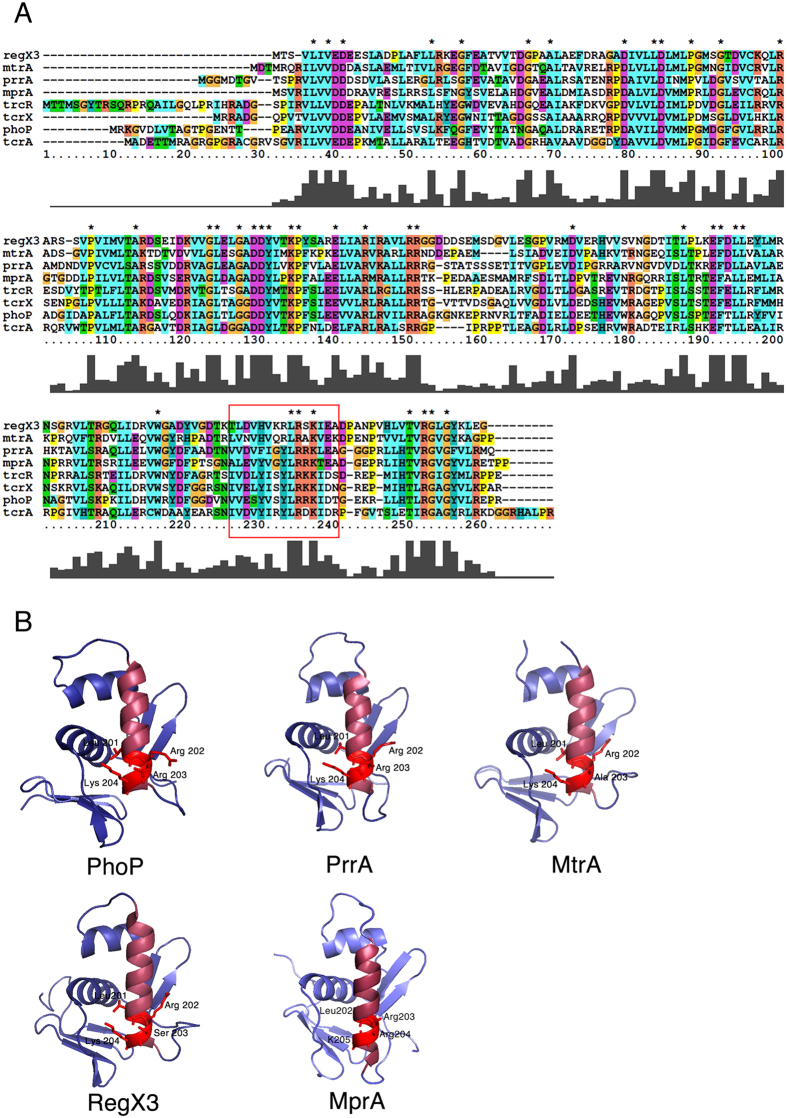
Multiple sequence alignment of response regulators of the OmpR family from Mtb and location of the LRXK motif derived from crystal structures or homology model of five response regulators from Mtb. (**A**) Sequences of 7 response regulators from Mtb that share structural homology with PhoP, were aligned using ClustalX2. The recognition helix is marked with a red border and the residues Leucine 201, Arginine 202 and Lysine 204 (Leucine202, Arginine 203 and Lysine 205 in the case of MprA) within the helix are indicated by *, which represents conserved residues. (**B**) Pymol representations of the crystal structures of the DNA binding domains of PhoP, PrrA, MtrA and RegX3 showing the recognition helices colored in raspberry and the LRXK residues as red sticks. The MprA structure shown is a homology model generated by Prime.

**Figure 2:**
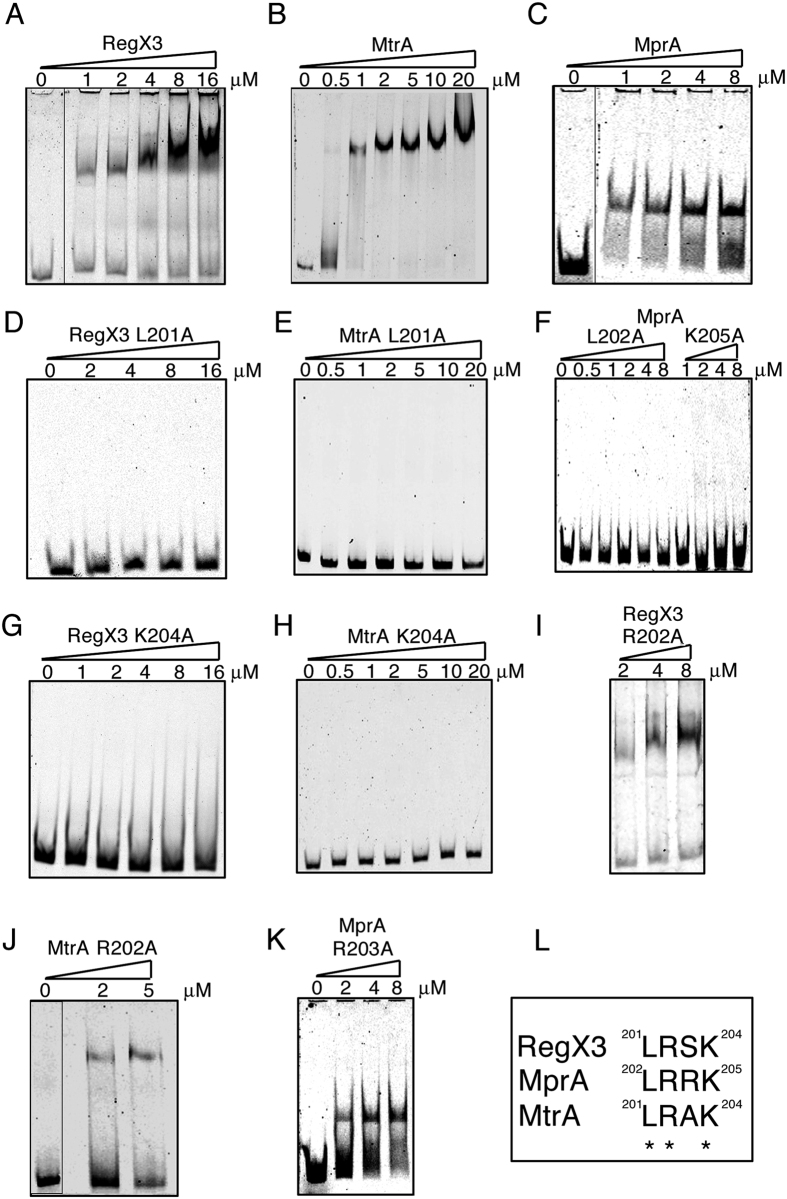
Leucine 201/202 and lysine 204/205 are essential for DNA binding activity of MtrA, RegX3 and MprA. Electrophoretic mobility shift assays of the DNA binding activity of RegX3 (**A**), MtrA (**B**) and MprA (**C**) or their mutants (**D**–**K**). Increasing concentrations of phosphorylated RegX3 or MtrA or MprA were incubated with Cy5-labelled DNA fragments from the *ppk1* or *fbpB* or *mprA* promoter respectively. The DNA-protein complexes were separated by 5% non-denaturing PAGE and visualized on a Typhoon imager.

Several RRs from Mtb have structural homology to PhoP, a response regulator of the OmpR family, especially at the C-terminal DNA binding domain^20^. Sequence alignments of eight such RRs from Mtb showed conservation of the motif LRXK at the C-terminal end (Fig. 1A) which was unique to this family of RRs. The positions of these residues in MtrA and RegX3 were: L201, R202, X203 (where X is A for MtrA and S for RegX3) and K204. In MprA, the residues were L202, R203, R204 and K205 (Fig. 1B). Since, two of these were positively charged (R202/203 and K204/205) the probability of direct interaction with the negatively charged DNA backbone was high^21^. To confirm the role of these residues in DNA binding, we chose three representative RRs of the OmpR family, MprA, RegX3 and MtrA and performed EMSAs using purified wild type (WT) or mutant proteins and their respective target promoter DNAs, *ppK1* (for RegX3)^21^, *mprA* (for MprA)^23^ and *fbpB* (for MtrA)^24^. Each WT protein bound to its target protein in a concentration-dependent manner (Fig. 2A–C). We observed that the mutations L201A in the case of RegX3 and MtrA (L202A for MprA) and K204A in the case of RegX3, MtrA (K205A for MprA) abolished the DNA binding activities of these RRs to their respective target DNA (Fig. 2D–H). However, the mutation R202A in the case of RegX3, MtrA (R203A for MprA) did not abolish DNA binding activity of these RRs (Fig. 2I–K). We further analyzed these interactions by surface plasmon resonance (SPR). Biotin-labelled DNA fragments were immobilized on streptavidin-coated CM5 surfaces and the WT or mutant proteins were allowed to flow over these surfaces. As expected, the WT or R202A (R203A for MprA) mutant proteins showed higher responses than the K204A or L201A (K205A or L202A for MprA) mutants (Supplementary Fig. 1). These results strengthened our contention that the LRXK motif (Fig. 2L) is essential to the DNA binding ability of this set of RRs from the OmpR family, *in vitro*. This motif is unique to the OmpR family and absent in the NarL family of RRs such as DosR.

### DNA binding mutants of RegX3 or MprA of Mtb are compromised in gene regulatory function

**Figure 3:**
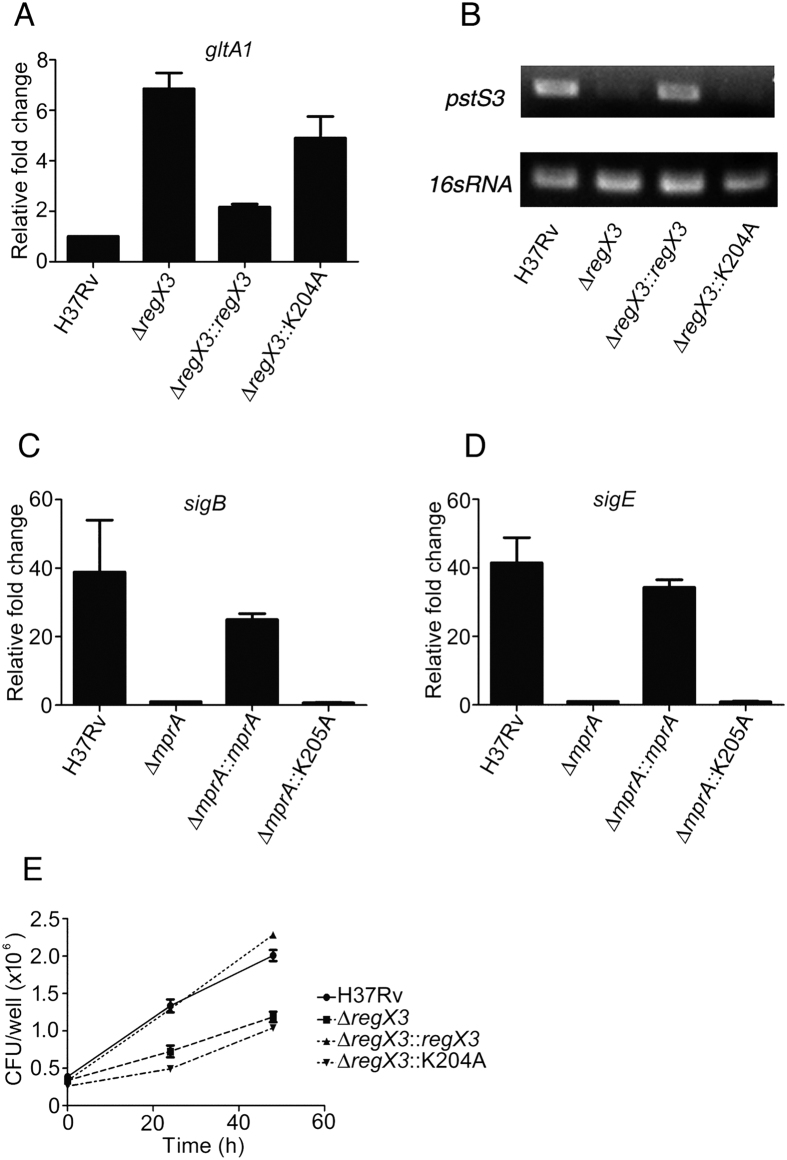
Lysine 204/205 is critical for RegX3 and MprA-dependent gene regulation in Mtb and survival of Mtb in macrophages. (**A,B**) *gltA1* or *pstS3* expression was evaluated by qRT-PCR (**A**) or semi-quantitative RT-PCR (**B**) after growth of the strains under nutrient-sufficient conditions (**A**) or under phosphate starvation (**B**). (**C,D**) *sigB* or *sigE* expression was evaluated by qRT-PCR after SDS treatment of cells as described in Methods. (**E**) RAW264.7 cells were infected with Mtb or Δ*regX3* or Δ*regX3*::*regX3* or Δ*regX3*::*regX3K204A* at an MOI of 10 and survival of Mtb was evaluated from CFUs. Data represent means ± SD (n = 3).

In order to investigate whether the role of the above amino acids extend to the ability of the RRs to regulate gene expression *in vivo*, we generated knockout strains of Mtb H37Rv in which the chromosomal copy of either *regX3* or *mprA* had been replaced by the hygromycin resistance gene (Supplementary Fig. 2a,b) using temperature sensitive mycobacteriophages^25^. Since *mtrA* is essential, it was not possible to inactivate this gene. The replacement of *regX3* or *mprA* by the hygromycin resistance gene in the respective mutant strains was confirmed by PCR (Supplementary Fig. 2c,d) and immunoblotting (Supplementary Fig. 2e,f). We complemented the *regX3* deletion strain (Δ*regX3*) with a copy of wild type *regX3* integrated into the chromosome under the control of its native promoter (Δ*regX3::regX3*). Compared to the wild type, Δ*regX3* showed upregulation of *gltA1* (or *prpC*) a RegX3-repressible gene^26^, under nutrition-sufficient conditions (Fig. 3A). This could be reversed by complementation with WT *regX3* but not with *regX3 K204A.* RegX3 regulates a distinct set of genes under phosphate starvation including *pstS3*^14^. The Δ*regX3* strain was compromised in its ability to induce the expression of *pstS3* under phosphate starvation. *regX3 WT* but not *regX3 K204A* complemented Δ*regX3* restoring *pstS3* expression (Fig. 3B). Taken together, these results suggested that the RegX3 K204A mutant is functionally compromised in terms of regulating RegX3-dependent gene expression in Mtb grown *in vitro*.

To further strengthen our argument that K204 (K205 for MprA) was likely to be a functionally important residue across a set of RRs, we extended our studies to analyzing gene expression under SDS stress attributable to MprA. The transcript levels of two sigma factors, *sigE* and *sigB*, both MprA targets^16^,^17^, were significantly reduced in the *mprA* mutant strain (Δ*mprA*). These levels could be restored in the mutant strain upon complementation with wild type *mprA* but not with *mprA K205A* (Fig. 3C,D). These observations reinforced the view that amino acid residues conserved in the DNA recognition helix of a set of RRs likely play an important functional role across this family of RRs.

### A DNA binding mutant of Mtb RegX3 is compromised for survival in macrophages

Based on the report that survival of the Δ*regX3* strain is compromised in macrophages^10^, we infected the murine macrophage like cell line RAW264.7 with each of the three strains containing variants of *regX3* described above. In concordance with previously published observations, we observed that in comparison to the parental strain, the survival of the Δ*regX3* strain was compromised by ~2 fold in RAW264.7. The observed growth defect was restored by complementation of the mutant with *WT regX3* but not *K204A regX3* (Fig. 3E). This suggested that the LRXK motif plays a role in bacterial survival in macrophages. Mutation of residue R202 to alanine did not compromise DNA binding ability of RegX3 (Fig. 2I). In harmony with this, the *ΔregX3::regX3R202A* strain showed growth characteristics similar to the wild type (Supplementary Fig. S2g).

### Targeting the LRXK motif by molecular docking

Taking into account that the conserved LRXK motif was critical for DNA binding and function of each RRs, we reasoned that an inhibitor docking to this region could potentially target multiple RRs of this family. We performed molecular docking studies to identify inhibitors that targeted this motif. We hypothesized that such inhibitors would compromise survival of Mtb both *in vitro* as well as *in vivo*.

The amino acids L201, R202 and K204 are present in the DNA binding domain of MtrA at the α7 helix loop end which is very near the protein’s inter-domain interface. In the activated state the α7 helix is required for interaction with DNA and the α7-α8 transactivation loop participates in an interaction with RNA polymerase^27^. Sitemapping of the MtrA crystal structure (PDB Id - 2GWR) identified a pocket close to the amino acids L201, R202 and K204. The selected sitemap was used for the generation of Grid1 (with the coordinates x = 37.4489, y = 3.0456 and z = 8.6644). The centroid of L201, R202 and K204 was selected as Grid2 (with coordinates x = 27.5382, y = −0.136 and z = 10.2869). These two grids (Supplementary Fig. 3a) were used to screen a BITS Pilani database for MtrA inhibitors. Similarly for RegX3, site mapping and grid generation was performed with its crystal structure (PDB Id - 2OQR)^28^. We identified a pocket close to the amino acids L201, R202 and K204 (Supplementary Fig. 3b). For MprA, the lack of a crystal structure required us to build a homology model using Prime (details of the method are provided in the supplemental section). This homology model was then used for site mapping and grid generation (Supplementary Fig. 3c).

**Figure 4:**
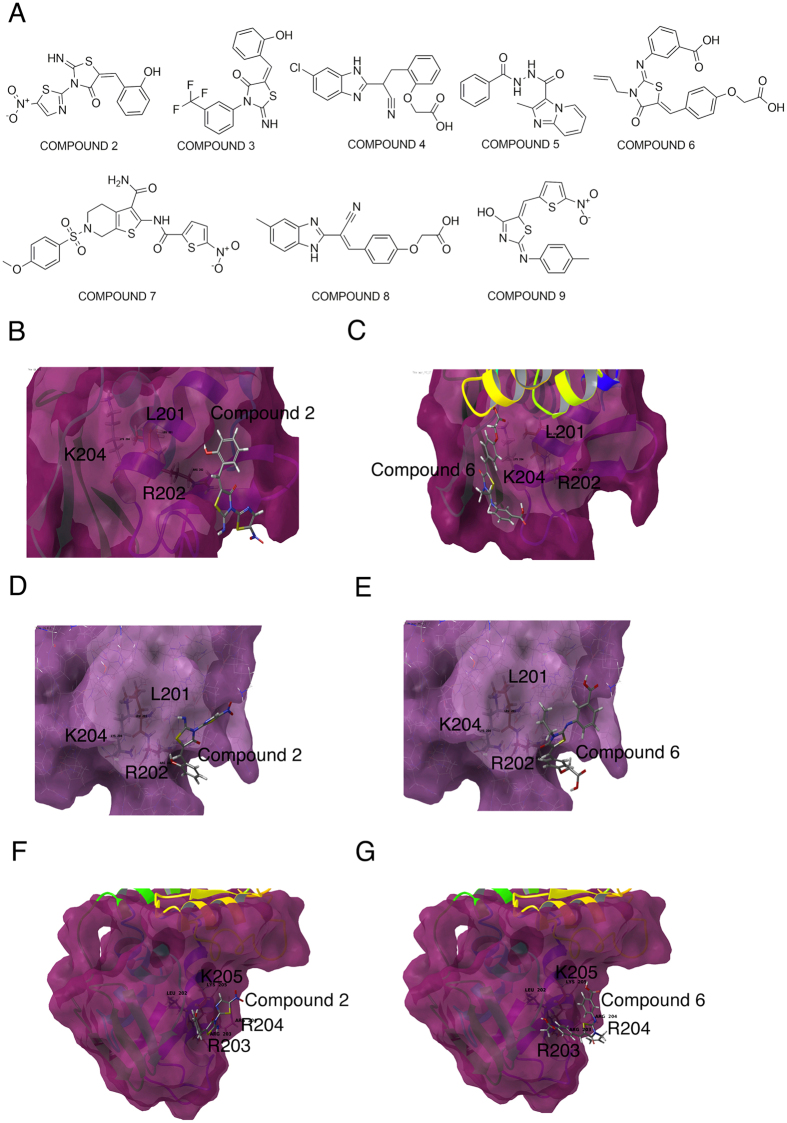
Structure-based identification of inhibitors of MprA, MtrA and RegX3. Site mapping of MtrA and RegX3 and the compounds that appear as positive hits. (**A**) Structures of compounds that were screened against MtrA. (**B–E**) Models of compounds 2 (**B,D,F**) or 6 (**C,E,G**) docked to MtrA (**B,C**), RegX3 (**D,E**) or MprA (**F,G**). The binding pockets around the L201, R202 and K204 residues (shown as sticks and labelled) are depicted. In case of MprA the residues are L202, R203 and K205.

**Figure 5:**
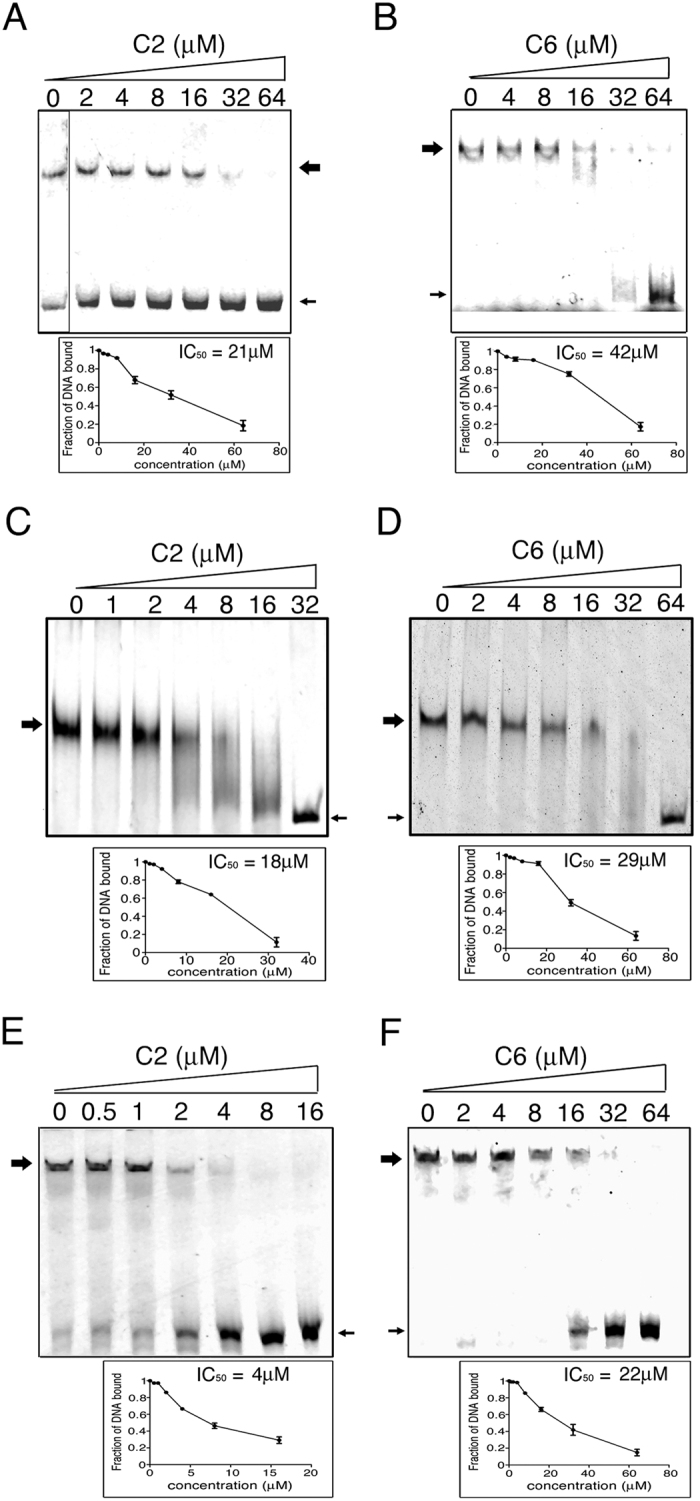
Compounds 2 (C2) and 6 (C6) inhibit DNA binding of MtrA, RegX3 and MprA *in vitro*. (**A,C,E**) C2 inhibits binding of MtrA (**A**), RegX3 (**C**) and MprA (**E**) to the *fbpB*, *ppk1* and *mprA* promoters respectively as assessed by EMSA. (**B,D,F**) C6 inhibits binding of MtrA (**A**), RegX3 (**C**) and MprA (**E**) to the *fbpB*, *ppk1* and *mprA* promoters respectively as assessed by EMSA. The plots beneath each electropherogram represent the respective inhibition curves with the IC_50_ values obtained after densitometry performed using a Typhoon imager. Large arrows indicate the positions of the DNA protein complexes. Small arrows indicate free DNA.

2500 compounds were initially docked to each grid in high throughput virtual screening (HTVS) mode and scored with glide scoring function. The top 20% scoring (∼500) molecules were re-docked using SP (shape and physiochemical properties) docking and the top 10% (∼100) were re-docked using XP (extra precision) docking. The hits obtained by XP had better G score values (−3.5 to −2.26 kcal/mol). The docked hits from the SP showed a sharp decrease in their G score values from −4.09 kcal/mol to almost −5 kcal/mol; after −2 kcal/mol the increase in G score value was significantly smaller. Therefore, the cut off G score value was chosen as −2 kcal/mol for docking hits of the XP run. Eight compounds (2–9) (Fig. 4A) showed G scores lower than the cut off (Supplementary Table 1). The compounds that showed up as hits against MtrA were also docked against the grids in RegX3 and MprA to check their docking energies. All the eight compounds docked at higher energies with RegX3 and MprA. The G scores were in the range of −1.7 kcal/mol to −2.9 kcal/mol (Supplementary Table 1). The eight inhibitors identified as high scoring hits that docked against the LRXK motifs in MtrA, RegX3 and MprA (Fig. 4A) were tested for their ability to inhibit DNA binding activity. Compound 2 [2IT4O or 2-iminothiazolidine-4-one] and Compound 6 [OTABA or oxo-1,3-thiazolidin-2-ylidene amino benzoic acid], (C2 and C6 respectively) could inhibit DNA binding activity of MtrA (Fig. 5A,B), whereas the other molecules were not effective (Supplementary Fig. 4a). C2 and C6 also inhibited the binding ability of RegX3 (Fig. 5C,D) and MprA (Fig. 5E,F). C2 was more effective than C6 (as apparent from the IC_50_ values shown in Fig. 5). The docking of C2 and C6 to the DNA binding pockets of MtrA, RegX3 and MprA (including the LRXK motif), is shown in Fig. 4(B–G). The inhibitory activity of C2 was specific for the OmpR family of RRs. C2 did not inhibit binding of DosR, a member of the NarL family to an *hspX* promoter-derived DNA fragment (Supplementary Fig. 4b).

### C2 and C6 inhibit the growth of Mtb *in vitro*

**Figure 6:**
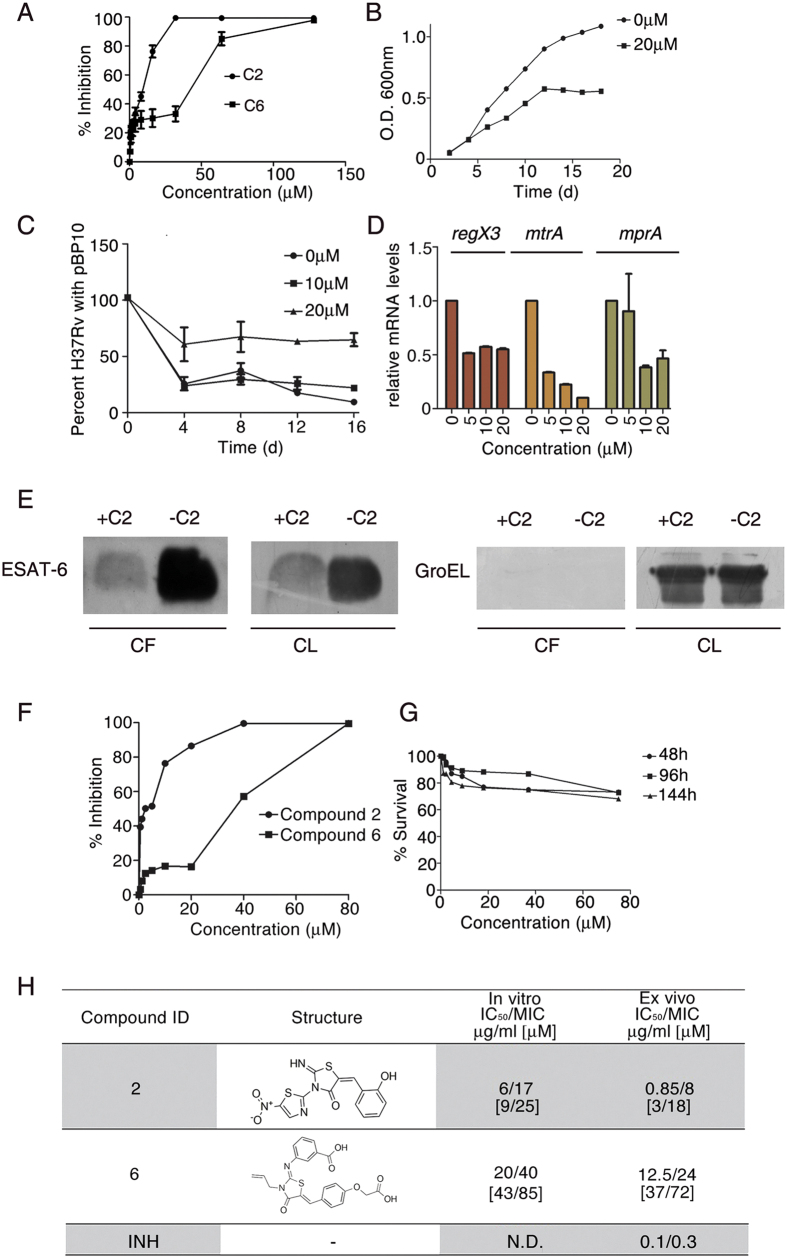
C2 Inhibits the growth of Mtb, the expression of *mtrA*, *regX3* and *mprA*, the secretion of ESAT-6 and the survival of Mtb in macrophages. (**A**) Alamar Blue survival assays for Mtb grown *in vitro* in the presence of C2 or C6. Means ± SD (n = 3). (**B**) Growth curve of Mtb in the presence of C2. (**C**) Retention of pBP10 by Mtb grown in the absence or presence of C2. Means ± SD (n = 3). Replication of Mtb was inhibited by C2. (**D**) Expression of *mtrA*, *regX3 and mprA* in Mtb grown in the presence of various concentrations of C2. Means ± SD (n = 3). Downregulation of all three RRs was observed. (**E**) Immunoblots of ESAT-6 and GroEL in Mtb Erdman culture filtrates (CF) and cell lysates (CL) obtained with (+) or without (−) C2 treatment. (**F**) Alamar Blue survival assays for Mtb in RAW264.7 in the presence of C2 or C6. Means ± SD (n = 3). (**G**) Survival of RAW264.7 in the presence of C2 was measured by Alamar Blue. (**H**) IC_50_ and MIC values of C2 and C6 against Mtb *in vitro* and in RAW264.7 (*ex vivo*). N.D., not determined.

Having established the ability of C2 and C6 to inhibit DNA binding of MtrA, RegX3 and MprA, we next determined the ability of these compounds to inhibit Mtb growth *in vitro* using Alamar Blue assays. We observed that the IC_50_ values of C2 and C6 against Mtb were 9 μM (~6 μg/ml) and 43 μM (~20 μg/ml), respectively (Fig. 6A,H). The efficacy of C2 was higher compared to that of C6. The lower efficacy of C6 was in harmony with its lesser ability to inhibit DNA binding (Fig. 5). The growth curve of Mtb in the presence of C2 levelled off earlier than growth in the absence of C2 (Fig. 6B). Considering that C2 restricted growth of Mtb, we assessed bacterial replication in its presence exploiting the unstable plasmid pBP10 which is lost at a quantifiable rate from dividing cells in the absence of antibiotic selection^29^. Interestingly, exposure to C2 resulted in higher levels of retention of pBP10 by Mtb after four days of growth in the absence of kanamycin (Fig. 6C), suggesting that C2 inhibits replication of Mtb *in vitro*, which agrees with the growth inhibition observed in the presence of C2.

### C2 regulates expression of *mtrA*, *regX3* and *mprA* and their targets

To confirm that C2 targets *mtrA*, *regX3* and *mprA* in Mtb cultures, we analyzed the expression of these autoregulated^23^,^24^,^30^ RRs in Mtb grown in the presence of C2. The expression of all three RRs was inhibited after exposure for 48 h to C2 (Fig. 6D). C2 expectedly regulated the expression of several targets of these three RRs. It augmented the expression of *rpfB* an MtrA-repressible target^31^, as well as *gltA1* and *cydA*, RegX3- repressible targets (Supplementary Fig. S4c,d). It repressed the expression of the MprA-activated targets *sigB*, *sigE* and *Rv0081*, the MtrA-activated targets *ftsI* and *dnaN*^24^ and the RegX3-activated target *pstS3* (Supplementary Fig. S4c,d) confirming that C2 inhibits the gene regulatory functions of MtrA, RegX3 and MprA.

### C2 inhibits the ESX-1 secretion apparatus

Mtb utilizes the type VII ESX-1 secretion apparatus to translocate substrates such as ESAT-6^32^. Secretion through this virulence-associated apparatus requires the unlinked *espACD* operon^33^. Transcription of *espACD* is regulated by EspR and EspR depletion leads to reduced bacterial survival^34^. Two OmpR family RRs, PhoP and MprA regulate *espR*^35^. In view of this, we tested the possibility that targeting of RRs by C2 impacts the expression of *espR* and the *espACD* operon. We observed that C2 reduced the expression of both *espA* and *espR* in Mtb after 48 hours of treatment (Supplementary Fig. S4e). In harmony with this, we observed reduced amounts of ESAT-6 in the culture filtrate of Mtb Erdman treated with C2 (Fig. 6E).

### C2 inhibits growth of Mtb in macrophages and augments the release of nitric oxide

As the next logical step, we tested the efficacy of C2 and C6 in infected macrophages. RAW 264.7 cells were infected with Mtb in the absence or presence of C2 or C6. Bacterial survival was assayed after 4 days. The IC_50_ values of C2 and C6 against Mtb grown in RAW264.7, were 3 μM (~0.85 μg/ml) and 37 μM (~12.5 μg/ml), respectively (Fig. 6F,H). C2 was more effective than C6 in infected macrophages. C2 did not affect the viability of RAW264.7 (Fig. 6G).

**Figure 7:**
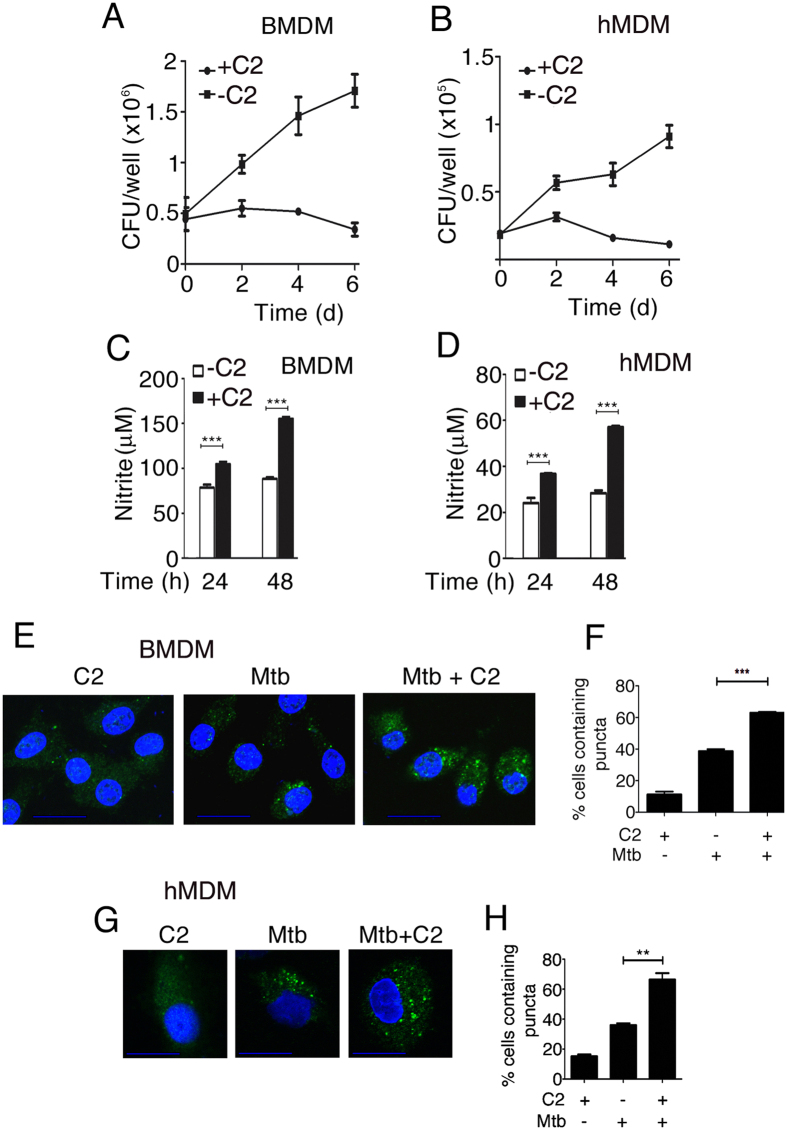
C2 inhibits Mtb Growth, induces NO release, and promotes autophagy in BMDMs and hMDMs. (**A,B**) BMDMs (**A**) or hMDMs (**B**) were infected with Mtb at an MOI of 5 and left in complete medium with (+C2) or without (−C2) C2 (20 μM) for 6 days. Bacterial CFUs were determined every 2 days in the presence or absence of C2. Means ± SD (n = 3). A significant reduction in bacterial load was observed in the presence of C2. (**C,D**) NO release by Mtb infected BMDMs (**C**) or hMDMs (**D**) upon treatment with C2 (20 μM). Means ± SD (n = 3); ***p ≤ 0.001. (**E,G**) Confocal microscopy of BMDMs (**E**) or hMDMs (**G**) treated with C2 (20 μM). Nuclei were stained with DAPI; green: LC3. (**F,H**) Quantification of puncta formation in BMDMs (**E**) or hMDMs (**G**) shown as the percentage of cells containing puncta. (Means ± SD, n = 3; **p ≤ 0.01, ***p ≤ 0.001). C2 promoted puncta formation.

In order to quantify the survival of Mtb in primary macrophages in the presence of C2, murine bone marrow derived macrophages (BMDM) or human monocyte derived macrophages (hMDM) were infected with Mtb at an MOI of 5. In BMDMs and MDMs we observed a ~8 fold decrease in CFU after 6 days in the presence of C2 (Fig. 7A,B). We argued that the effects of a compound on Mtb in infected macrophages depend not only on its ability to regulate bacterial gene expression programs, but also on associated changes in the immune response during infection. Considering that the survival of Mtb in macrophages is diminished in the presence of C2, we tested for the production of the antibacterial effector NO. NO release from infected macrophages was significantly higher in the presence of C2 than in its absence (Fig. 7C,D; Supplementary Fig. S5a). C2 alone did not affect NO release from uninfected macrophages (data not shown).

### C2 augments autophagy

We further reasoned that apart from augmented NO release, enhanced lysosomal trafficking of Mtb would also inhibit the survival of Mtb in the presence of C2. Autophagy enhances lysosomal trafficking of Mtb^36^. We therefore tested whether C2 impacts autophagy in Mtb-infected macrophages. The conversion of LC3-I to LC3-II is an established marker of autophagy. This was enhanced in Mtb infected RAW 264.7 cells in the presence of C2 (Supplementary Fig. S5b).

Autophagy is also characterized by formation of LC3 puncta. We confirmed the effect of C2 by enumerating the formation of LC3 puncta (by immunostaining) in infected macrophages. Puncta formation was enhanced in the presence of C2 (Fig. 7E–H; Supplementary Fig. S5c,d) as in the case of isoniazid (Supplementary Fig. S5e,f). Similar results were obtained in the presence of bafilomycin A an inhibitor of autophagic flux (data not shown). C2 therefore augments the immune response by enhancing autophagy during Mtb infection.

## Discussion

Tuberculosis is usually treatable with a combination of four first-line antitubercular drugs. However, poor compliance has led to the emergence of multidrug resistant (MDR) and extensively drug resistant (XDR) organisms. The need to develop alternate therapeutic strategies is therefore obvious. The TCSs allow bacteria to sense and respond to changes in the environment, including host-mediated antimicrobial activities. As sensors of environmental signals, TCSs regulate diverse physiological processes such as sporulation, the equilibrium between a dormant and an actively growing state, utilization of nutrient sources such as carbon, nitrogen and phosphate, antibiotic resistance and competence^7^. They are functionally regulated by accessory proteins positioning them as hubs in networks of cellular information flow^37^. Coupled with the fact that they are absent in eukaryotes, the TCSs represent attractive drug targets. Mtb encodes several members of the OmpR family of RRs which share a common winged helix-turn-helix motif in their output domain. Here we focused on three representative RRs, MtrA, RegX3 and MprA. MtrA is the only known essential RR of Mtb. It regulates genes associated with DNA replication, cell division and cell wall biosynthesis^24^. SenX3-RegX3 responds to phosphate starvation, and a *regX3* transposon mutant is attenuated in the lungs of mice and guinea pigs following low dose aerosol infection^14^. RegX3 regulates genes that are involved in energy metabolism, cell envelope maintenance and regulatory functions^10^. *mprA-mprB* is upregulated in the lungs of mice during infection^38^, and also in an artificial hollow-fiber granuloma model^39^. This TCS modulates ESX-1 function^40^ which is a prototype of type VII secretion systems of Gram-positive bacteria.

We argued that based on the conservation of amino acid sequence in the DNA recognition helix of the OmpR family of RRs of Mtb, we could conceivably target all three of the above RRs simultaneously. Furthermore it is documented that inactivation of TCSs cause attenuation as in the case of Mtb *ΔphoPR*^41^,^42^ and Mtb *ΔprrAB*^15^. Multiple sequence alignments of the DNA binding domains of the OmpR family of RRs in Mtb showed the presence of a conserved LRXK motif in their recognition helix. We tested our hypothesis that this motif is required for the DNA binding activities of MtrA, RegX3 and MprA by EMSAs and SPR-based protein-DNA interaction analyses. For each of the three RRs, mutating the conserved amino acid residues L201/202 or K204/205, compromised DNA binding ability, supporting our hypothesis (Fig. 2). In order to establish the role of the aforesaid amino acids *in vivo*, molecular genetic manipulations were carried out to inactivate the chromosomal copies of two (RegX3 and MprA) of these RRs. As expected, we observed that Δ*regX3* and Δ*mprA* strains were highly compromised in terms of their ability to regulate genes known to be part of the RegX3 and MprA regulons respectively. In order to ascertain whether mutants carrying substitutions of the critical amino acid residues in the DNA recognition region could restore the functions of these RRs *in vivo*, we complemented each knockout strain with either the wild type or the respective DNA binding mutants of *regX3* or *mprA*. As expected, the mutated copies of the respective proteins could not appropriately regulate the expression of downstream genes (Fig. 3). We sought to target this domain of high conservation such that a single molecule might simultaneously inhibit multiple Mtb TCSs.

Target based approaches have been associated with high attrition rates^43^. Here we have taken as a starting point, lead structures obtained from whole cell screens to search a library of compounds developed at BITS Pilani with known anti-mycobacterial activity. The crystal structure of one of the representative RRs was used as a template in energy-based pharmacophore modelling and in silico docking to the DNA binding pocket. Eight molecules were selected for further analysis, of which two, C2 and C6 both based on a thiazolidine scaffold, could effectively inhibit the three RRs chosen by us. Here we show that the 2-imino thiazolidine-4-one derivative, C2 has higher efficacy in terms of inhibition of DNA binding activity of MtrA, MprA and RegX3 (as evaluated by EMSA), and lower MIC compared to C6 (Fig. 6). We therefore took up C2 for further detailed studies. For Mtb grown *in vitro*, C2 could inhibit the transcription of all three of the aforesaid RRs, and modulate downstream genes regulated by these RRs. It is logical to assume that C2 will also target other OmpR family RRs PrrA and PhoP and their regulons, since both these RRs also contain the LRXK motif.

The type VII ESX-1 secretion system is encoded by the *esx-1* genetic locus, and is essential for the export of two major virulence factors of Mtb, namely ESAT-6 (EsxA) and CFP-10 (EsxB)^32^. The secretion of ESX-1 substrates also requires the products of the *espACD* operon. EspR is a transcriptional regulator that activates the transcription of *espA, espC* and *espD*^34^. Recent studies have linked MprAB and PhoPR to the expression of EspR^35^ and the regulation of the *esx-1* region^40^. Based on these reports, we hypothesized that C2 could potentially regulate *espR* transcription. The downregulation of *espR* in the presence of C2 confirmed our hypothesis. In harmony with this, we observed that C2 inhibits the expression of *espA*. Pang *et al.*^40^ have reported that MprAB regulates the ESX-1 region, whereas Rybnicker *et al.*^44^ have reported that a compound which deregulates MprAB, affects the ESX-1 region and the ESX-1 dependent secretion of ESAT-6. Taking together the fact that C2 inhibits MprA and that secretion of ESAT-6 depends on products of the *esx-1* and the *espACD* operons, we tested the secretion of ESAT-6. For this purpose, we took Mtb Erdman as the strain of choice, since Mtb H37Rv shows limited secretion of ESAT-6^45^. We observed reduced secretion of ESAT-6 in the culture filtrate of C2-treated Mtb Erdman cells (Fig. 6E). In addition, C2 treatment lowered *esxA* expression and lower ESAT-6 was observed in the lysates of C2-treated cells. The mechanism of reduced expression of *esxA*, remains unclear at present. Reduction in *esxA* expression and a defective secretion machinery could together contribute to the diminished amounts of ESAT-6 in the culture filtrate of C2-treated cells. ESAT-6 is a central molecule in host-pathogen interactions. For example, it promotes pathogen survival and dissemination by arresting phagosome maturation and facilitating necrosis. Defective production and secretion of ESAT-6 is therefore likely to have wide-ranging implications in the context of infection.

The environment of Mtb within its host is associated with oxygen depletion, nutrient stress, oxidative and nitrosative stress. Mtb must therefore be capable of efficiently sensing the environment and reprogramming its transcriptome in order to maximise its chance of enduring within this hostile environment by dampening the host immune response. C2 was capable of regulating gene expression attributable to each of the three chosen RRs. We reasoned that an impaired bacterial signal transduction machinery would most likely influence the response of macrophages to infection. For example, it has been reported that an *mprAB* knockout strain of Mtb elicits diminished levels of TNF-α and IL-1β from macrophages, compared to the wild type Mtb^40^. We tested whether C2 alters the host response to Mtb infection. The innate immune response requires the participation of macrophages, dendritic cells, epithelial cells, neutrophils and other immune cells culminating in events that augment cellular antimicrobial mechanisms, reduce inflammation and go on to shape the adaptive immune response. In this study we restricted ourselves to analysing the effects of C2 on infected macrophages. Autophagy has been associated with degradation of long-lived proteins to provide energy under nutritional stress and in removing misfolded or aggregated proteins or damaged organelles. In addition it plays a pivotal role in defense against intracellular pathogens^46^,^47^. Activation of the ubiquitin-mediated autophagy pathway results in reduced survival of Mtb in macrophages. The first-line antimycobacterial drug isoniazid activates autophagy^48^. With this background information, we tested the effect of C2 on Mtb-mediated autophagy in murine BMDM, hMDM and RAW264.7. Here we present evidence that C2 triggers enhanced autophagy during Mtb infection. Using a Western blot assay for LC3-II formation and fluorescence microscopy for enumeration of LC3 puncta, we observed enhanced autophagy in the presence of C2 early during infection. This effect was apparent even in the presence of bafilomycin A, confirming that the observed increase in LC3-II in the presence of C2 was not due to a block in autophagic flux. In addition, C2 also augmented release of NO in Mtb-infected macrophages (Fig. 7). C2 regulated bacterial pathways conceivably alter bacterial processes in a manner that influences the host immune response. While it is possible that C2 could exert effects on mycobacterial targets other than the RRs, it is tempting to speculate that simultaneous incapacitation of multiple RRs could be directly associated with enhanced autophagy. Taken together our results demonstrate that (a) DNA binding activity of multiple TCSs depends on a conserved motif which can be targeted to modulate stress responsive genes associated with the respective RRs; (b) a compound that docks to this DNA binding pocket can effectively inhibit multiple RRs simultaneously; and (c) targeting multiple RRs by a small molecule can twist the host-pathogen interaction to alter autophagy and NO production with a concomitant loss of bacterial burden in macrophages. The results described here serve as a proof of principle that TCSs can be simultaneously targeted to strike down pathways important for bacterial survival and tilt the balance of the host-pathogen interaction in favor of the host. It opens up new avenues of exploration in our quest for a new drug against TB. In the case of Mtb, resistance to drugs usually occurs through chromosomal mutations rather than through the acquisition of extrachromosomal resistance determinants or through mobile genetic elements. Resistance to a single drug requires mutation in a single gene. Combination chemotherapy makes the emergence of a spontaneous mutant resistant to all the components of the regimen, less likely. By this same token, we suggest that resistance to a drug capable of targeting multiple RRs would be far less likely, since it would necessitate mutations of multiple genes. This approach therefore appears to be an attractive option for development of new drugs.

## Methods

### Bacterial strains, media and growth conditions

Mycobacterial strains (H37Rv and Erdman) were routinely maintained in Middlebrook 7H9 medium supplemented with 10% ADC or OADC. Unless mentioned specifically, Mtb refers to the H37Rv strain. Detailed bacterial growth conditions have been provided in the supplemental section.

### Cell culture

RAW 264.7 cells were routinely maintained in DMEM with 10% FBS, 4 mM glutamine and penicillin/streptomycin. Human MDMs were derived from peripheral blood monocytes. BMDMs were obtained from C57BL/6 mice. Detailed cell culture methods have been provided in the supplemental section.

### Construction of Mtb mutants and plasmids

Mtb knock out mutants of *mprA* and *regX3* were generated using temperature sensitive mycobacteriophages as described by Bardarov *et al.*^25^ Detailed methods for the generation of knock out mutants and complementation of these mutants with either wild type or mutant *mprA* or *regX3* are available in the supplemental section. A list of plasmids and bacterial strains is available on request.

### Expression and purification of recombinant proteins

RegX3, MprA, MtrA and SenX3 (lacking the 78 bp transmembrane domain) were amplified by PCR, cloned and over expressed in *E. coli.* DosR (DevR) expression construct was obtained from Prof. Jaya Tyagi, All India Institute of Medical Sciences, New Delhi. Mutant proteins were generated by site directed mutagenesis using overlap extension PCR. Detailed methods for purification of recombinant proteins are given in the supplemental section. A list of primers is provided with the supplemental “Methods”.

### Quantitative real time PCR

Total RNA was extracted from bacterial cultures, reverse transcribed and Real time PCR was performed on an Applied Biosystems 7500 machine using MesaGreen master mix (Eurogentec) following the manufacturer’s instruction. A list of primers is provided with the supplemental “Methods”.

### Quantification of bacterial growth and replication

Mtb growth was monitored by following change in absorbance at 600 nm. Alternatively, Alamar blue assay was used. Detailed method has been provided in the supplemental section.

### Surface Plasmon Resonance analysis of protein-DNA binding

Double stranded DNA was biotin labelled and immobilised on a streptavidin coated sensor chip. Wild type or mutant proteins were injected at different concentrations and binding response was measured in a BIAcore X-100 instrument (GE Healthcare). Detailed method has been provided in supplemental section.

### *In vitro* phosphorylation and electrophoretic mobility shift (EMSA) assays

Autophosphorylation of MprB or SenX3 was carried out in the presence of 16 mM ATP followed by incubation with purified MprA or RegX3 for transphosphorylation. MtrA was phosphorylated using EnvZ as described by Sharma *et al.*^31^. DosR was phosphorylated using acetyl phosphate. For EMSAs, phosphorylated MtrA or RegX3 or MprA or DosR was incubated with 5′-Cy5- or ^32^P- labelled DNA and bound complex was separated on 5–6% non denaturing PAGE followed by detection using a Typhoon Trio Plus Imager (GE Healthcare). Detailed methods have been provided in the supplemental section.

### Docking studies

Crystal structures of RegX3 (PDB Id 2OQR) and MtrA (PDB Id 2GWR) were obtained from PDB and energy minimised using the OPLS 2005 forcefield in Schrodinger suite version 9.3. A homology model of MprA was developed using Prime and the crystal structures of PrrA and PhoP (PDB Ids 3ROJ, 1YS7, 1YS6, 1KGS, 3F6P, 2ZWM and 1NXO) as templates. The model was energy minimised using the OPLS 2005 forcefield in Schrodinger suite version 9.3. Active site prediction was done using the Sitemap module while grid generation was done using the Glide module of Schrodinger LLC version 3.5. 2500 compounds in the BITS database were then docked against the generated sitemaps and grids by high throughput virtual screening (HTVS). Shortlisted compounds were used for further studies. Detailed methods have been provided in the supplemental section.

### Survival of Mtb in macrophages

Macrophages were grown and infected with Mtb for 4 h. Cells were lysed at the indicated times and viable bacteria were enumerated.

### Culture filtrate preparation and Western blotting

Mtb Erdman culture filtrates were obtained after growing bacteria in Sauton’s media. Proteins were immunoblotted with ESAT-6 or GroEL antibody (obtained through BEI Resources, NIH, NIAID). Detailed methods have been provided in the supplemental section.

### Autophagy assays

For autophagy analysis, infected macrophages were lysed and immunoblotted with LC3 antibody. For the visualisation of LC3 puncta, infected macrophages were incubated with LC3 antibody followed by immunostaining with Alexa 488-conjugated secondary antibody. Images were acquired in a Leica confocal microscope. At least 100 cells were counted to enumerate the number of punctated cells. Detailed methods have been provided in the supplemental section.

### Nitrite assays

For NO (nitrite) measurements, RAW264.7 was infected with Mtb in DMEM for 24–48 h. Nitrite was measured using Griess reagent (Invitrogen), according to the manufacturer’s instructions. Absorbance was read at 540 nm in an ELISA reader and the concentrations of nitrite were calculated against a standard curve.

### Ethics statement

Animal experiments were approved by the institutional Animal Ethics Committee of the Bose Institute, Kolkata, India and were carried out in accordance with the approved guidelines.

### Statistical analysis

Statistical analyses were performed using GraphPad Prism 5 software. Data have been expressed as means ± SD. Two-tailed Student *t* test was used when comparing two groups. *p < 0.05; **p < 0.01; ***p < *0.001*.

## Additional Information

**How to cite this article**: Banerjee, S. K. *et al.* Targeting multiple response regulators of *Mycobacterium tuberculosis* augments the host immune response to infection. *Sci. Rep.* **6**, 25851; doi: 10.1038/srep25851 (2016).

## Supplementary Material

Supplementary Information

## References

[b1] Lechartier B., Rybniker J., Zumia A. & Cole S. T. Tuberculosis drug discovery in the post-post-genomic era. EMBO Mol. Med. 6, 158–168 (2014). 24401837 10.1002/emmm.201201772 PMC3927952

[b2] Betts J. C., Lukey P. T., Robb L. C., McAdam R. A. & Duncan K. Evaluation of a nutrient starvation model of *Mycobacterium tuberculosis* persistence by gene and protein expression profiling. Mol. Microbiol. 43, 717–731 (2002). 11929527 10.1046/j.1365-2958.2002.02779.x

[b3] Fisher M. A., Plikaytis B. B. & Shinnick T. M. Microarray analysis of the *Mycobacterium tuberculosis* transcriptional response to the acidic conditions found in phagosomes. J. Bacteriol. 184, 4025–4032 (2002). 12081975 10.1128/JB.184.14.4025-4032.2002 PMC135184

[b4] Via L. E. *et al.* Tuberculous granulomas are hypoxic in guinea pigs, rabbits, and nonhuman primates. Infect. Immun. 76, 2333–2340 (2008). 18347040 10.1128/IAI.01515-07 PMC2423064

[b5] Voskuil M. I. *Mycobacterium tuberculosis* gene expression during environmental conditions associated with latency. Tuberculosis (Edinb) 84, 138–143 (2004). 15207483 10.1016/j.tube.2003.12.008

[b6] Wayne L. G. & Hayes L. G. An *in vitro* model for sequential study of shiftdown of *Mycobacterium tuberculosis* through two stages of nonreplicating persistence. Infect. Immun. 64, 2062–2069 (1996). 8675308 10.1128/iai.64.6.2062-2069.1996 PMC174037

[b7] Krell T. *et al.* Bacterial sensor kinases: diversity in the recognition of environmental signals. Annu. Rev. Microbiol., 64, 539–559 (2010). 20825354 10.1146/annurev.micro.112408.134054

[b8] Stock A. M., Robinson V. L. & Goudreau P. N. Two-component signal transduction. Annu. Rev. Biochem. 69, 183–215 (2000). 10966457 10.1146/annurev.biochem.69.1.183

[b9] Bretl D. J., Demetriadou C. & Zahrt T. C. Adaptation to environmental stimuli within the host: two-component signal transduction systems of *Mycobacterium tuberculosis*. Microbiol. Mol. Biol. Rev. 75, 566–582 (2011). 22126994 10.1128/MMBR.05004-11 PMC3232741

[b10] Parish T., Smith D. A., Roberts G., Betts J. & Stoker N. G. The senX3-regX3 two-component regulatory system of *Mycobacterium tuberculosis* is required for virulence. Microbiology 149, 1423–35 (2003). 12777483 10.1099/mic.0.26245-0

[b11] Galperin M. Y. Structural classification of bacterial response regulators: diversity of output domains and domain combinations. J. Bacteriol. 188, 4169–4182 (2006). 16740923 10.1128/JB.01887-05 PMC1482966

[b12] Martínez-Hackert E. & Stock A. M. The DNA-binding domain of OmpR: crystal structures of a winged helix transcription factor. Structure 5, 109–124 (1997). 9016718 10.1016/s0969-2126(97)00170-6

[b13] Walters S. B. *et al.* The *Mycobacterium tuberculosis* PhoPR two-component system regulates genes essential for virulence and complex lipid biosynthesis. Mol. Microbiol. 60, 312–330 (2006). 16573683 10.1111/j.1365-2958.2006.05102.x

[b14] Rifat D., Bishai W. R. & Karakousis P. C. Phosphate depletion: a novel trigger for *Mycobacterium tuberculosis* persistence. J. Infect. Dis. 200, 1126–1135 (2009). 19686042 10.1086/605700

[b15] Haydel S. E., Malhotra V., Cornelison G. L. & Clark-Curtiss J. E. The prrAB two-component system is essential for *Mycobacterium tuberculosis* viability and is induced under nitrogen-limiting conditions. J. Bacteriol. 194, 354–361 (2012). 22081401 10.1128/JB.06258-11 PMC3256671

[b16] He H., Hovey R., Kane J., Singh V. & Zahrt T. C. MprAB Is a stress-responsive two-component system that directly regulates expression of sigma factors SigB and SigE in *Mycobacterium tuberculosis*. J. Bacteriol. 188, 2134–2143 (2006). 16513743 10.1128/JB.188.6.2134-2143.2006 PMC1428128

[b17] Pang X. *et al.* Evidence for complex interactions of stress-associated regulons in an mprAB deletion mutant of *Mycobacterium tuberculosis*. Microbiology 153, 1229–1242 (2007). 17379732 10.1099/mic.0.29281-0

[b18] Fol M. *et al.* Modulation of *Mycobacterium tuberculosis* proliferation by MtrA, an essential two-component response regulator. Mol. Microbiol. 60, 643–657 (2006). 16629667 10.1111/j.1365-2958.2006.05137.x

[b19] Zahrt T. C. & Deretic V. An essential two-component signal transduction system in *Mycobacterium tuberculosis*. J. Bacteriol. 182, 3832–3838 (2000). 10851001 10.1128/jb.182.13.3832-3838.2000 PMC94557

[b20] Menon S. & Wang S. Structure of the response regulator PhoP from *Mycobacterium tuberculosis* reveals a dimer through the receiver domain. Biochemistry 50, 5948–5957 (2011). 21634789 10.1021/bi2005575 PMC3133661

[b21] Mizuno T. & Tanaka I. Structure of the DNA-binding domain of the OmpR family of response regulators. Mol. Microbiol. 24, 665–670 (1997). 9179858 10.1046/j.1365-2958.1997.3571723.x

[b22] Sanyal S., Banerjee S. K., Banerjee R., Mukhopadhyay J. & Kundu M. Polyphosphate kinase 1, a central node in the stress response network of *Mycobacterium tuberculosis*, connects the two-component systems MprAB and SenX3–RegX3 and the extracytoplasmic function sigma factor, sigma E. Microbiology 159, 2074–2086 (2013). 23946493 10.1099/mic.0.068452-0

[b23] He H. & Zahrt T. C. Identification and characterization of a regulatory sequence recognized by *Mycobacterium tuberculosis* persistence regulator MprA. J. Bacteriol. 187, 202–212 (2005). 15601704 10.1128/JB.187.1.202-212.2005 PMC538824

[b24] Rajagopalan M. *et al.* *Mycobacterium tuberculosis* origin of replication and the promoter for immunodominant secreted antigen 85B are the targets of MtrA, the essential response regulator. J. Biol. Chem. 285, 15816–15827 (2010). 20223818 10.1074/jbc.M109.040097 PMC2871449

[b25] Bardarov S. *et al.* Specialized transduction: an efficient method for generating marked and unmarked targeted gene disruptions in *Mycobacterium tuberculosis*, *M. bovis* BCG and *M. smegmatis*. Microbiology 148, 3007–3017 (2002). 12368434 10.1099/00221287-148-10-3007

[b26] Roberts G., Vadrevu I. S., Madiraju M. V. & Parish T. Control of CydB and GltA1 expression by the SenX3 RegX3 two component regulatory system of *Mycobacterium tuberculosis*. PLoS ONE. 6, e21090 (2011). 21698211 10.1371/journal.pone.0021090 PMC3116866

[b27] Friedland N. *et al.* Domain orientation in the inactive response regulator *Mycobacterium tuberculosis* MtrA provides a barrier to activation. Biochemistry 46, 6733–6743 (2007). 17511470 10.1021/bi602546q PMC2528954

[b28] King-Scott J. *et al.* The structure of a full-length response regulator from *Mycobacterium tuberculosis* in a stabilized three-dimensional domain-swapped, activated state. J. Biol. Chem. 282, 37717–37729 (2007). 17942407 10.1074/jbc.M705081200

[b29] Gill W. P. *et al.* A replication clock for *Mycobacterium tuberculosis*. Nat. Med. 15, 211–214 (2009). 19182798 10.1038/nm.1915 PMC2779834

[b30] Himpens S., Locht C. & Supply P. Molecular characterization of the mycobacterial SenX3–RegX3 two-component system: evidence for autoregulation. Microbiology 146, 3091–3098 (2000). 11101667 10.1099/00221287-146-12-3091

[b31] Sharma A. K. *et al.* MtrA, an essential response regulator of the MtrAB two component system regulates the transcription of resuscitation promoting factor B (RpfB) of *Mycobacterium tuberculosis*. Microbiology. 161, 1271–1281 (2015). 25833257 10.1099/mic.0.000087

[b32] Abdallah A. M. *et al.* Type VII secretion – mycobacteria show the way. Nat. Rev. Microbiol. 5, 883–891 (2007). 17922044 10.1038/nrmicro1773

[b33] Houben E. N. G., Korotkovc K. V. & Bitter W. Take five – Type VII secretion systems of mycobacteria.Biochim. Biophys. Acta 1843, 1707–1716 (2014). 10.1016/j.bbamcr.2013.11.003 24263244

[b34] Raghavan S., Manzanillo P., Chan K., Dovery C. & Cox J. S. Secreted transcription factor controls *Mycobacterium tuberculosis* virulence. Nature 454, 717–722 (2008). 18685700 10.1038/nature07219 PMC2862998

[b35] Cao G. *et al.* EspR, a regulator of the ESX-1 secretion system in *Mycobacterium tuberculosis*, is directly regulated by the two-component systems MprAB and PhoPR. Microbiology 161, 477–489 (2015). 25536998 10.1099/mic.0.000023

[b36] Alonso S., Pethe K., Russell D. G. & Purdy G. Lysosomal killing of *Mycobacterium* mediated by ubiquitin-derived peptides is enhanced by autophagy. Proc. Natl. Acad. Sci. USA 104, 6031–6036 (2007). 17389386 10.1073/pnas.0700036104 PMC1851611

[b37] Jung K., Fried L., Behr S. & Heermann R. Histidine kinases and response regulators in networks. Current Opinion in Microbiology 15, 118–124 (2012). 22172627 10.1016/j.mib.2011.11.009

[b38] Talaat A. M., Lyons R., Howard S. T. & Johnston. S. A. The temporal expression profile of *Mycobacterium tuberculosis* infection in mice. Proc. Natl. Acad. Sci. USA 101, 4602–4607 (2004). 15070764 10.1073/pnas.0306023101 PMC384793

[b39] Karakousis P. C. *et al.* Dormancy phenotype displayed by extracellular *Mycobacterium tuberculosis* within artificial granulomas in mice. J. Exp. Med. 200, 647–657 (2004). 15353557 10.1084/jem.20040646 PMC2212740

[b40] Pang X. *et al.* MprAB regulates the espA operon in *Mycobacterium tuberculosis* and modulates ESX-1 function and host cytokine response. J. Bacteriol. 195, 66–75 (2013). 23104803 10.1128/JB.01067-12 PMC3536182

[b41] Gonzalo-Asensio J. *et al.* PhoP: a missing piece in the intricate puzzle of *Mycobacterium tuberculosis*. PLos one 3, e3496 (2008). 18946503 10.1371/journal.pone.0003496 PMC2566814

[b42] Johnson B. K. *et al.* The carbonic anhydrase inhibitor ethoxzolamide inhibits the *Mycobacterium tuberculosis* PhoPR regulon and Esx-1 secretion and attenuates virulence. Antimicrob. Agents Chemother. 59, 4436–4445 (2015). 25987613 10.1128/AAC.00719-15 PMC4505220

[b43] Payne D. J., Gwynn M. N., Holmes D. J. & Pompliano D. L. Drugs for bad bugs: confronting the challenges of antibacterial discovery. Nature Reviews Drug Discovery 6, 29–40 (2007). 17159923 10.1038/nrd2201

[b44] Rybniker J. *et al.* Anticytolytic screen identifies inhibitors of mycobacterial virulence protein secretion. Cell Host & Microbe 16, 538–548 (2014). 25299337 10.1016/j.chom.2014.09.008

[b45] Solans L. *et al.* A specific polymorphism in *Mycobacterium tuberculosis* H37Rv causes differential ESAT-6 expression and identifies WhiB6 as a novel ESX-1 component. Infect. Immun. 82, 3446–3456 (2014). 24891105 10.1128/IAI.01824-14 PMC4136221

[b46] Deretic V., Saitoh T. & Akira S. Autophagy in infection, inflammation and immunity. Nature Rev. Immunol. 13, 722–737 (2013). 10.1038/nri3532 PMC5340150 24064518

[b47] Fabri M., Realegeno S. E., Jo E.-K. & Modlin R. L. Role of autophagy in the host response to microbial infection and potential for therapy. Curr. Opin. Immunol. 23, 65–70 (2011). 21071195 10.1016/j.coi.2010.10.010 PMC3042547

[b48] Kim J.-J. *et al.* Host cell autophagy activated by antibiotics is required for their effective antimycobacterial drug action. Cell Host & Microbe 11, 457–468 (2012). 22607799 10.1016/j.chom.2012.03.008

