# Supplementary material for: Targeting multiple response regulators of Mycobacterium tuberculosis augments the host immune response to infection
Source: Sci Rep. 2016 May 16;6:25851. doi: 10.1038/srep25851 (PMC4867592; doi:10.1038/srep25851)
Supplement: Supplementary Information [file srep25851-s1.pdf]

SUPPLEMENTARY INFORMATION

Title: Targeting multiple response regulators of *Mycobacterium tuberculosis* augments the host immune response to infection

Authors: Srijon Kaushik Banerjee[1], Manish Kumar[1], Reshma Alokam[2] , Arun Kumar Sharma[1], Ayan Chatterjee[1], Ranjeet Kumar[1], Sanjaya Kumar Sahu[1], Kuladip Jana[3], Ramandeep Singh[4], Perumal Yogeeswari[2], Dharmarajan Sriram[2], Joyoti Basu[1,5] and Manikuntala Kundu[1,5,6]

Affiliations: [1]Department of Chemistry, Bose Institute, 93/1 Acharya Prafulla Chandra Road, Kolkata 700009, India,

[2]Department of Pharmacy, Birla Institute of Technology & Science-Pilani, Hyderabad Campus, Jawahar Nagar, Hyderabad 500078, India

[3]Division of Molecular Medicine, Bose Institute, P-1/12 CIT Scheme VII M, Kolkata 700054, India

[4]Vaccine and Infectious Disease Research Centre, Translational Health Science and Technology Institute, NCR-Biotech Science Cluster, 3rd Milestone, Faridabad Gurgaon Expressway. Faridabad-121001, India

[5]Co senior authors

[6]For correspondence

E-mail: manikuntala@vsnl.net

Supplementary Figure 1

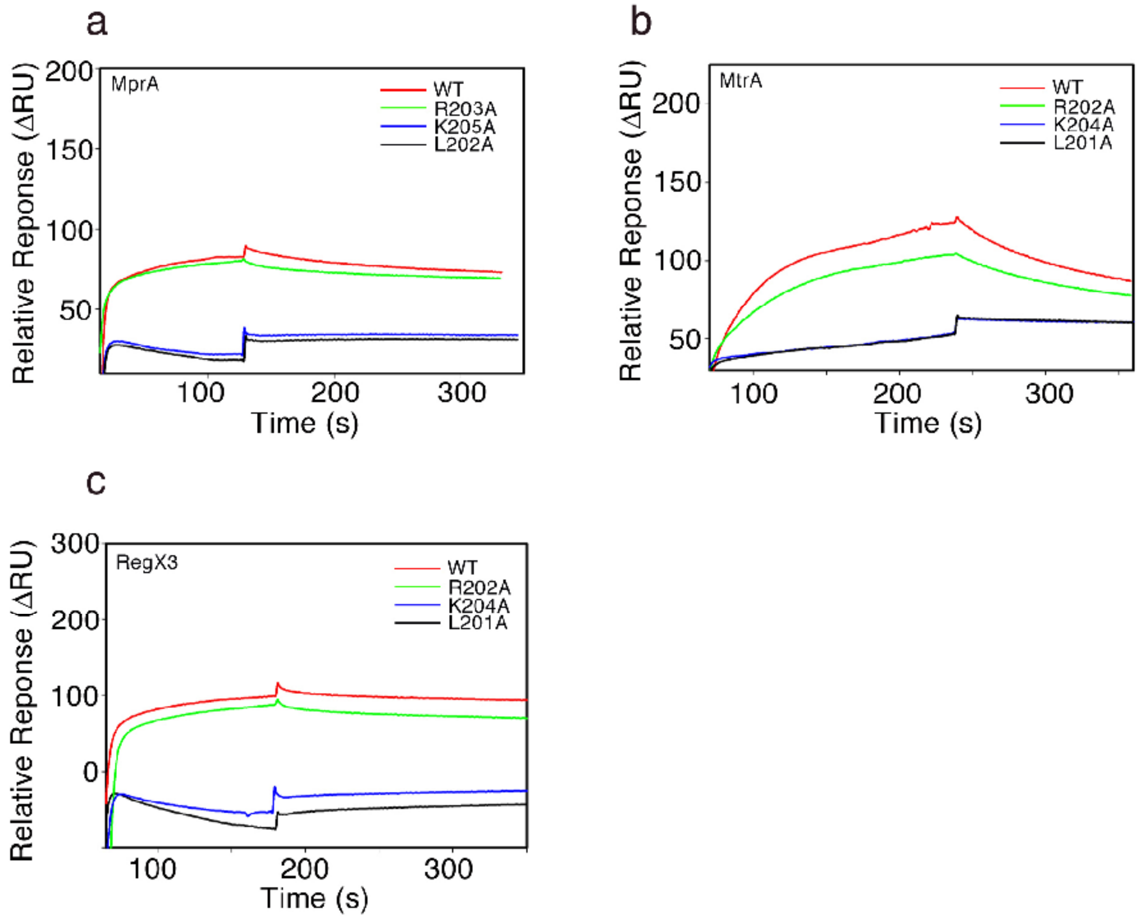

Supplementary Figure 1. Surface plasmon resonance analysis of the binding of wild type or mutant RRs to their respective DNA. (**a-c**) Promoter DNAs labelled at the 5' ends with biotin were immobilised on streptavidin-coated surfaces. The purified proteins MtrA or Regx3 or MprA (or their mutants) were run over the respective immobilised DNA and changes in RU were detected.

Supplementary Figure 2.

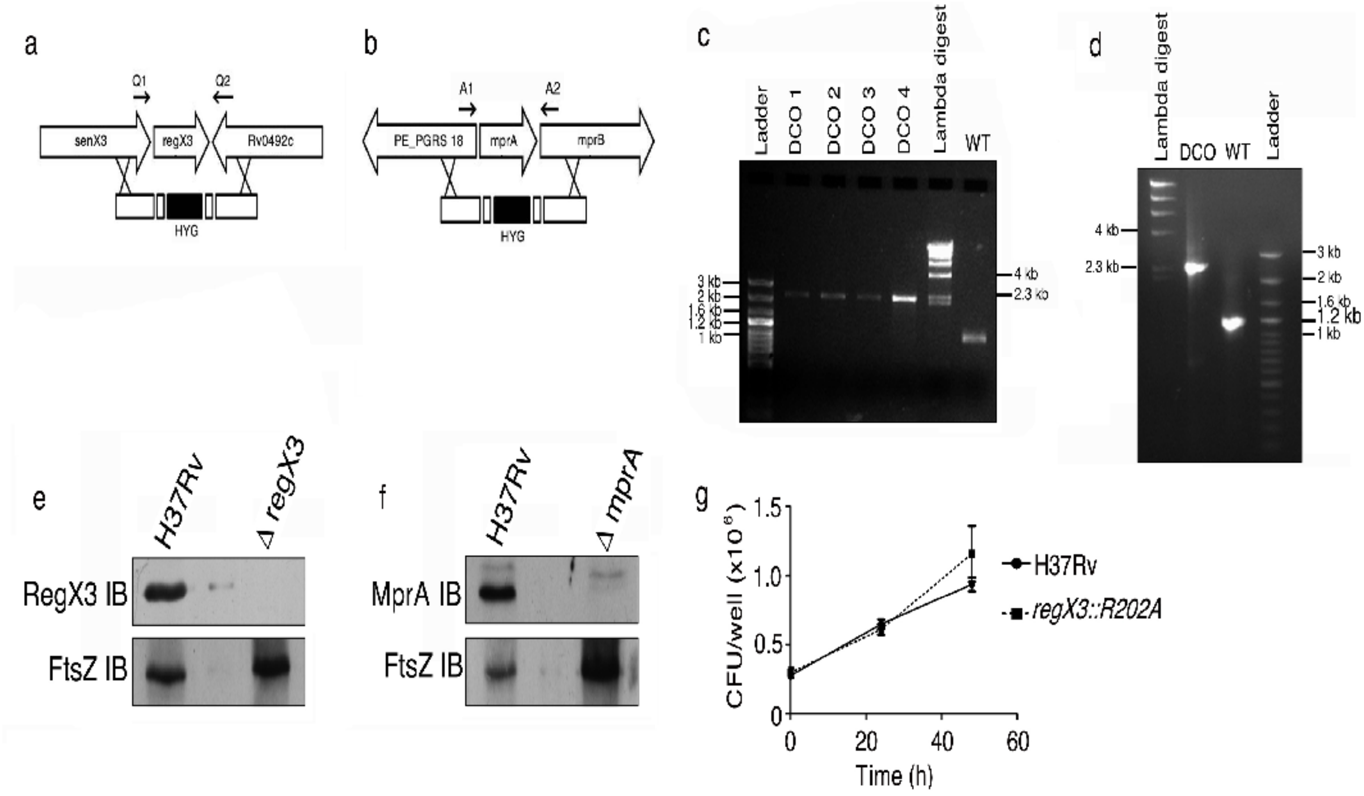

Supplementary Figure 2.Strategy for knock out of *regX3* and *mprA* and confirmation of knockouts. (**a**) Organisation of the genomic region containing *regX3* and a representation of the knockout construct containing the *Hyg* gene. Primers Q1 and Q2 were used to confirm the knockout by PCR. (**b**) Organisation of the genomic region containing *mprA* and a representation of the knockout construct containing the *Hyg* gene. Primers A1 and A2 were used to confirm the knockout by PCR. Desired amplicons were 1.1 kb for the wild type and 2.3 kb for the knockout mutants of *regX3* and *mprA*respectively. (**c,d**) Generation of *regX3* (**c**) or *mprA* (**d**) knockout was confirmed by PCR using primers described in panels A and B. Four clones (DCOs 1 to 4) were found to be positive in the case of the *regX3* knockout mutant, and one for the *mprA* knockout mutant. (**e,f**) Knock out of *regX3* (**e**, using DCO4) and *mprA* (**f**) was confirmed by Western blotting using RegX3 or MprA antibody respectively. FtsZ antibody was used to confirm equal loading.

Supplementary Figure 3.

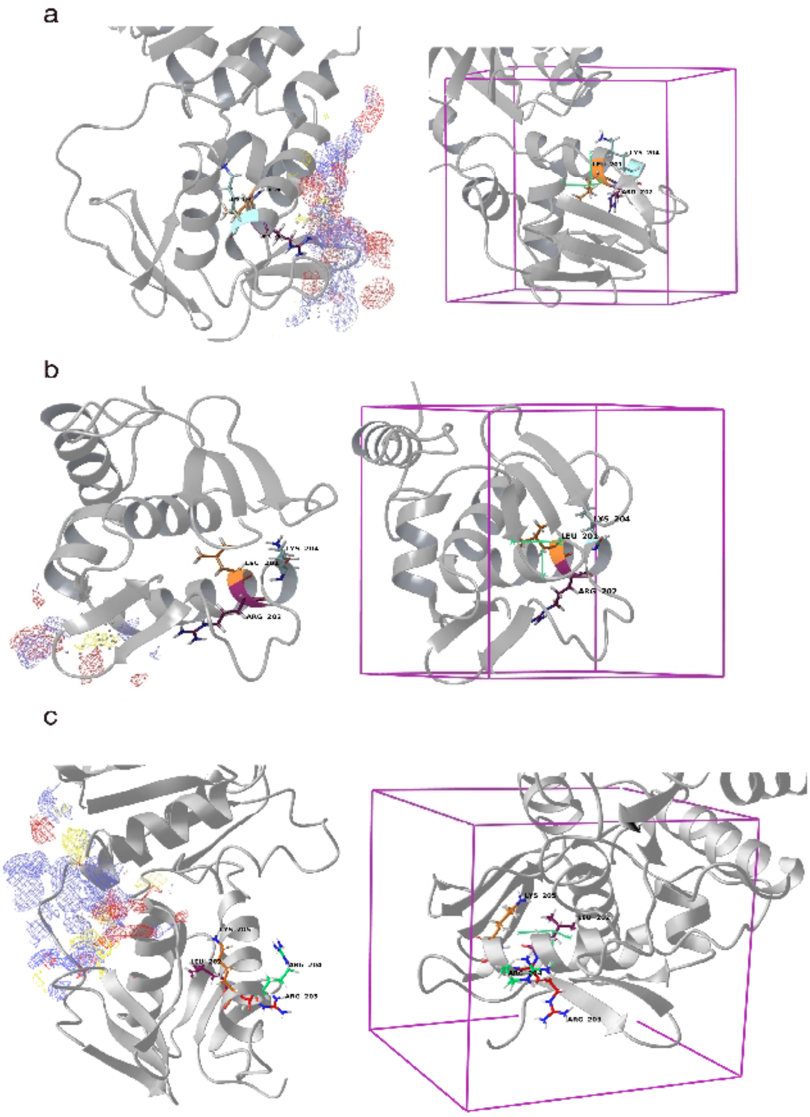

Supplementary Figure 3. Sitemapping and grid formation on MtrA, RegX3 and MprA. Panels on the left: MtrA (**a**), RegX3 (**b**) and MprA (**c**) sitemapping shows the mesh area indicating the binding pocket for docking the compound library. Panels on the right: Grid formation based on the centroid of the three amino acids L201, R202 and K204 in MtrA (**a**) or RegX3 (**b**) or the amino acids L202, R203 and K205 in MprA (**c**).

Supplementary Figure 4.

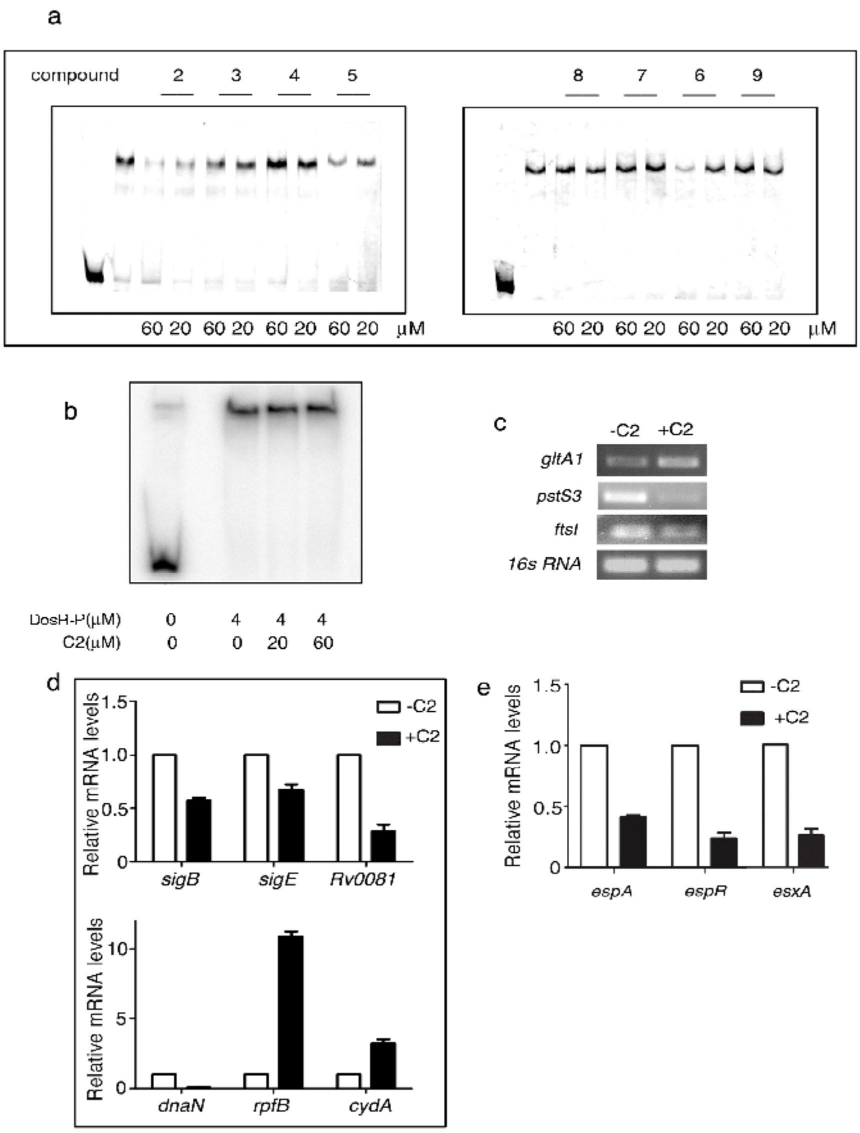

Supplementary Figure 4. Effects of compounds on DNA binding ability and transcription of genes regulated by MtrA, RegX3, MprA and DosR (DevR). (**a,b**) Electrophoretic mobility shift assays of the DNA binding ability of (**a**) MtrA with Cy5-labelled DNA fragment from *fbpB* or (**b**) DosR (DevR) with ^{32}P-labelled DNA fragment from *hspX* were carried out in the absence or presence of compounds 2 to 9 (**a**) or compound 2 (**b**) as indicated. The first lanes of each panel represent free oligo. (**c,d**) *M. tuberculosis* was grown in the absence (-) or presence of C2 (+) (20 μM) and transcription of *gltA1*, *pstS3*, *ftsI*, *sigB*, *sigE, Rv0081, dnaN, cydA* or *rpfB,* was evaluated by semi-quantitative RT-PCR (**c**) or qRT-PCR (**d**). (**e**) Expression of *espA*, *espR* and *esxA* after treatment with C2 (20μM).

Supplementary Figure 5

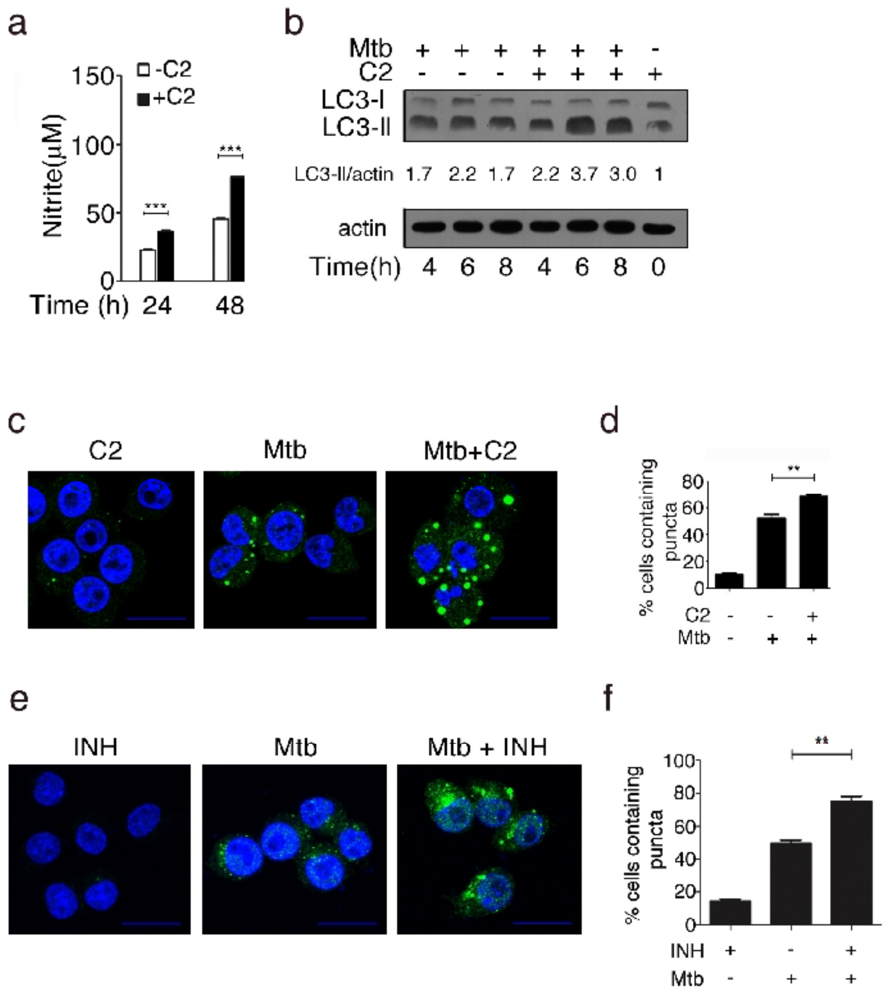

Supplementary Figure 5. C2 induces NO release and promotes puncta formation. (**a**) RAW 264.7 cells were infected with Mtb at an MOI of 10 and treated with C2. NO release was measured from the cell free supernatants after 24 or 48 hours. (**b**) RAW 264.7 cells were infected with Mtb at an MOI of 10 and the effect of C2 on autophagy was evaluated by assessing the conversion of LC3-I to LC3-II by immunoblotting. Densitometric analysis was performed to quantify the conversion. C2 promoted conversion of LC3-I to LC3-II. (**c-f**) Confocal microscopy of infected RAW 264.7 cells after treatment with C2 (**c,d**) or with isoniazid (0.5 µg/ml) (positive control) (**e,f**). Immunostaining was done using LC3 antibody, nuclei were stained with DAPI. Puncta formation was quantified as percentage of cells containing puncta. C2 promotes puncta formation. **p $\leq$ 0.01 ($\pm$ SD, n=3).

Table S1. Interacting energies of the compounds selected for testing in the in silico glide module using the MtrA, RegX3 and MprA grids.

Protein	Compound no.	H Bond	Glide G score	Docking score
MtrA	2	3	-3.75957	-3.75957
	3	5	-3.48213	-3.48213
	4	3	-3.35551	-3.35551
	5	3	-3.96962	-3.96962
	6	5	-3.77394	-3.77394
	7	3	-3.31824	-3.31824
	8	4	-2.76614	-2.76614
	9	4	-2.60866	-2.60866
RegX3	2	4	-1.817	-1.817
	3	3	-2.887	-2.887
	4	2	-2.087	-2.087
	5	2	-1.725	-1.725
	6	2	-2.835	-2.835
	7	4	2.655	2.655
	8	1	-1.962	-1.962
	9	1	-2.212	-2.212
MprA	2	3	-2.288	-2.288
	3	3	-1.703	-1.703
	4	2	-1.702	-1.702
	5	5	-1.926	-1.926
	6	3	-2.233	-2.233
	7	2	-2.033	-2.033
	8	1	-1.541	-1.541
	9	3	-1.939	-1.939

Supplemental Methods

Bacterial strains and growth

E. coli Top10 and DH5α were used for cloning. *E. coli* BL21(DE3), C41(DE3) and C43(DE3) (a kind gift from Prof. J. S. Tyagi) were used for recombinant protein expression. *E. coli* was grown in Luria-Bertani (LB) Miller broth or on LB agar (Becton Dickinson, Difco) at 37°C. *Mycobacterium tuberculosis* (Mtb) H37Rv or Mtb Erdman or other genetically manipulated strains were grown in Middle Brook (MB) 7H9 (Difco) broth supplemented with 10% v/v OADC (Difco), 0.05% Tween 80 (Hi Media Laboratories, India) and appropriate antibiotics where required, at 37°C with shaking at 120 rpm. MB7H10 or 7H11 agar (Difco) supplemented with 10% v/v OADC (and antibiotics where required), was used as solid medium for plating Mtb strains. For *E. coli*, kanamycin sulfate (Roche Applied Science), ampicillin (Sigma), chloramphenicol (Sigma) and hygromycin were used at concentrations of 50, 100, 35 and 50 μg/ml respectively. For Mtb, kanamycin and hygromycin were used at concentrations of 20 and 50 μg/ml respectively.

Antibodies

Peptide-specific antibodies against Mtb MprA and RegX3 were raised by Thermo Fisher Scientific (Pierce). LC3B antibody for immunoblotting was from Cell Signaling Technology, USA; and LC3B antibody for immunofluorescence was from MBL Inc. HRP-conjugated secondary antibodies were from Cell Signaling Technology; Alexa 488-conjugated goat anti-rabbit IgG for microscopy was from Abcam. The following reagents were obtained through BEI Resources, NIAID, NIH: polyclonal anti-*Mycobacterium tuberculosis* ESAT6 (Gene Rv3875) (antiserum, rabbit), NR-13803 and monoclonal anti-*M. tuberculosis* GroEL2 (Gene Rv0440) clone IT-56 (CBA1) (produced *in vitro*), NR-13655.

Cloning, expression and purification of recombinant proteins

RegX3/Rv0491 684bp and SenX3/Rv0490 (truncated version lacking the 78bp transmembrane region) were cloned and purified as reported earlier (Sanyal *et al*., 2013). MprA/Rv0981 687bp was PCR amplified from Mtb H37Rv genomic DNA using the primer pair P5 and P6 (this and all other primers are given in the list of oligonucleotides) and cloned between the BamHI and KpnI sites of pACYC Duet-1(Novagen) to obtain pMprA. The construct was verified by sequencing and transformed into *E.coli* BL21(DE3). Induction was carried out at 16°C overnight with 0.5 mM IPTG. Cells were

lysed in 50 mM Tris-HCl pH8.0, containing 0.2% (v/v) Triton X 100, DNase (5 µg/ml), lysozyme (2 mg/ml) and 1X protease inhibitor cocktail (Roche Applied Science). His-MprA was purified from the cell-free lysate by Ni^{2+}-NTA affinity chromatography, concentrated (using a Sartorius 10 kDa MWCO membrane filter) and stored in 5% (v/v) glycerol. MprB/Rv0982 (1359bp; excluding the first 51 amino acids containing the transmembrane domain) was PCR amplified from Mtb genomic DNA using primer pair P7 and P8 and cloned between the BamHI and HindIII sites of pET29a+(Novagen) to obtain pMprB. pMprB was transformed into BL21(DE3). Protein was induced at 37°C for 3 h with 100 µM IPTG. Cells were lysed by sonication in 10 mM Tris-HCl pH 7.4, containing 1mM $MgCl_2$ and 1X protease inhibitor cocktail. Inclusion bodies were isolated by fractionation and stored at -80°C. S-MprB was obtained by extraction of inclusion bodies with 10 mM Tris-HCl pH 8.0 containing 3M guanidine hydrochloride (Gdn-HCl), 1 mM DTT and 1 mM EDTA for 30 minutes at 4°C with gentle shaking. The extract was centrifuged and the supernatant was subjected to dialysis against 100 mM Tris-HCl pH 8.0, containing 1 mM EDTA, 20% glycerol at 4°C for 12 h to remove Gdn-HCl. The dialysate was stored at -80°C until further use. Full length MtrA/Rv3246c excluding the stop codon was amplified using the primer pair P9-P10 and cloned between the NdeI and HindIII sites of pET20b+(Novagen) to generate *mtrA*::pET20b+. The construct was then transformed into *E. coli* C41(DE3). His-MtrA was induced using 500 µM IPTG at 16^0C for 20 h. Cells were harvested and resuspended in 20 mM Tris-HCl, pH 7.5 containing 5% glycerol and 0.7% 2-mercaptoethanol and lysed in buffer containing 2mg/ml lysozyme, 0.2% v/v Triton X-100, protease inhibitor cocktail and 5 µg/ml DNase I (Sigma). The cell-free supernatant was purified on Ni^{2+}-NTA agarose (Qiagen). Protein was concentrated using a Pierce protein concentrator (9kDa MWCO) and stored at -80^0C for further use.

Recombinant DosR (DevR) was purified as stated by Vashisht *et al.* (2016). Briefly, DosR His6-pET28a (a kind gift from Prof. Jaya Tyagi) was expressed in *E. coli* C43(DE3) cells at 25°C for 16 h with 1mM IPTG. The cells were sonicated in sodium phosphate buffer pH7.5 containing 50 mM NaCl. The cell-free supernatant was purified on Ni^{2+}-NTA agarose. Purified DevR was dialysed against sodium phosphate buffer pH 8 containing 50 mM NaCl and 5% glycerol, concentrated and stored at

-80°C in 50% glycerol.

Generating the alanine substitution mutants of the response regulators

All alanine substitution mutants were generated by overlap extension PCR. Briefly, two sets of primers were used for each mutation, with one set (e.g. P11, P12) containing the desired mutation and the other set (e.g. P1, P2) flanking the region of mutation. For generation of the RegX3 L201A mutant, two reactions were performed with primer pairs P11-P2 and P12-P1. The PCR products with overlaps were used as template for a final round of PCR with primer pair P1-P2. The product containing the desired mutation was cloned as described above and sequenced. Other mutants were generated similarly. All primers used for site directed mutagenesis are listed in the list of oligonucleotides.

In vitro autophosphorylation and transphosphorylation reactions

The phosphorylation of RegX3 and its mutants by SenX3 was carried out as described by Sanyal *et al*. (2013). Phosphorylation of MprA and its mutants was done as follows. S-MprB dialysate was incubated with S-agarose resin (Novagen) in binding buffer (20 mM Tris-HCl pH 7.5 containing 150 mM NaCl) for 40 minutes at 4°C with rocking. The resin was washed using 10 volumes of binding buffer followed by washing with 5 volumes of kinase buffer (50 mM Tris-HCl pH 7.6, 50 mM KCl and 50 mM $MgCl_2$). Resin bound S-MprB was autophosphorylated with 16.5 mM cold ATP in kinase buffer for 10 minutes at room temperature. Phospho-MprB was then incubated with purified His-MprA or its mutants for transphosphorylation at 37°C for 30 minutes. The phosphorylated proteins were then used for EMSA. For MtrA phosphorylation, EnvZ kinase (a kind gift from Prof. Michelle Igo, University of California, Davis) was phosphorylated in the presence of 20 mM cold ATP in 50 mM Tris HCl pH 8, containing 50 mM KCl, and 50mM $MgCl_2$ for 15 min at 37^oC. Transphosphorylation was carried out in kinase buffer with 2.5 µM phosphorylated EnvZ and His-MtrA or its Ala mutants. Phosphorylated MtrA and its mutants were then used for EMSA.

DosR phosphorylation was performed by incubating DosR with 50 mM acetyl phosphate (Sigma) in the presence of 40 mM Tris-HCl pH 8 and 5 mM $MgCl_2$ for 20 minutes at 25°C.

Electrophoretic mobility shift assays

Binding of RegX3, MprA, MtrA or their mutants to the respective DNA elements was analysed by EMSA. Binding of phospho-RegX3 (or its mutants) to the *ppK1* promoter was analysed as reported previously by Sanyal *et al*. (2013). MprA binding to the *mprA* promoter region was analyzed as described by He and Zahrt (2004). A 140 bp region, containing the MprA binding sites from the *mprA* promoter was PCR amplified using the primer pair P33-P34, and the resulting Cy5 labelled product was purified. 10 ng of the Cy5 labelled product was incubated with varying concentrations of phospho-MprA (or its mutants) in 4 mM Tris-HCl pH 8.0, containing 4 mM $MgCl_2$, 5% (v/v) glycerol, 40 mM NaCl, 0.5 µg poly(dI-dC) and 0.5 µg salmon sperm DNA, for 30 minutes at room temperature. The reaction mix was run on a 5-6% TBE polyacrylamide gel and the DNA-protein complex was detected using a Typhoon Trio Plus Imager (GE Healthcare). The binding of phospho-MtrA to the *fbpB* promoter (reported by Rajagopalan *et al*., 2010), was analysed by EMSA. A 147 bp region upstream of *fbpB* was PCR amplified using the primer pair P37-P38. Gel purified Cy5-labelled DNA was incubated with purified phospho-MtrA or its mutants in 20mM Tris/HCl, pH 8, containing 20 mM NaCl, 50 mM $CaCl_2$, 10 mM $MgCl_2$, 10 mM KCl, 5% (v/v) glycerol, 0.05 µg/µl salmon sperm DNA for 30 min at room temperature. Samples were separated on 6% TAE polyacrylamide gels (100V, 4°C for 90 min) and the DNA protein complex was visualized using a Typhoon Trio Plus Imager (GE Healthcare).

Binding of DosR to the *hspX* promoter-derived DNA fragment was analysed by labelling the DNA with ^{32}P-ATP. The labelled DNA was incubated with phosphorylated DosR for 20 minutes at 30°C in 25mM Tris-HCl pH 8, 6 mM $MgCl_2$, 20 mM KCl, 0.5 mM EDTA and 5% glycerol. The reactions were subjected to electrophoresis on a 6% non denaturing gel at 120V at 4°C.

Surface Plasmon Resonance

A CM5 surface (GE Healthcare) was activated by EDC and NHS injections followed by injection of streptavidin (50 µg/ml) in HBS-P (GE Healthcare). Ethanolamine was used to block the surface. 5' Biotin labeled promoter DNA was then injected for 180 seconds at a concentration of 50 ng/ml so that 150 RU of DNA was immobilized. Purified (RegX3 or MprA or MtrA, or mutant protein) was then run over this surface for 180 seconds and allowed to dissociate for 240 seconds. The surface was regenerated to baseline with 1 M NaCl and the next sample was injected. Each promoter-derived DNA fragment was generated using 5' biotin labeled versions of primers P33-P38 for PCR amplification from genomic DNA. Each DNA was purified and then used for SPR assays.

Generation of Δ*regX3*, Δ*mprA* and the various complemented strains in H37Rv

Mtb H37Rv strains lacking response regulators *regX3* or *mprA* were generated using temperature sensitive mycobacteriophages (Bardarov *et al.*, 2002). Briefly, ~800bp flanking regions upstream and downstream of *regX3* or *mprA* were PCR amplified using primers P39-P46 listed in the table of primers and cloned into the pYUB854 vector at sites flanking the hygromycin cassette. Each construct was then packaged into temperature sensitive mycobacteriophages and delivered via infection into H37Rv cells. Double crossovers (DCOs) were screened after 6 weeks by PCR and knockouts confirmed by western blot using antibodies against RegX3 or MprA. Complementation by wild type *regX3* or *mprA* was done as follows. A ~2.5 kb region encompassing the *senX3-regX3* promoter, and the *senX3* and *regX3* ORFs was PCR amplified from H37Rv genomic DNA using primer pair P49-P50 and cloned into the pMV306Kan vector. A ~900bp *senX3* region was deleted using Stu1 and the *regx3*pMV306 construct was electroporated into the *ΔregX3* strain to obtain *ΔregX3CT*. The *mprA*B region was complemented by cloning a PCR amplified (primer pair P47-P48) ~2.5 kb region containing the native *mprAB* promoter and the *mprA* and *mprB* ORFs into the pMV306Kan vector. The *mprAB*pMV306 construct was electroporated into the Δ*mprA* strain to obtain Δ*mprACT*. Mutations in the *mprA* and *regX3* genes were incorporated into the complementation constructs using the method described above.

Growth under phosphate starvation and SDS stress

For phosphate starvation, Mtb H37Rv or its variants were grown in MB7H9 containing MOPS pH 6.6, 17.6 mM Na_2HPO_4, 7.35 mM KH_2PO_4 and 0.05% Tween 80, till $O.D._{600}$ reached 0.6. The cells were then centrifuged and washed twice with phosphate-free MB7H9, resuspended in either phosphate-containing media or phosphate-free media and allowed to grow at 37°C at a shaking speed of 120 rpm for 72 h (Sanyal *et al.*, 2013). Aliquots were removed and cells were stored in RNA Later (Qiagen) at -80°C for isolation of RNA, or as pellets for cell-free lysate preparation. For SDS stress, Mtb H37Rv or its variants were grown in MB7H9 till $O.D._{600}$ was equal to 0.2-0.3 followed by addition of 0.05% SDS. The bacteria were grown in SDS for 5 hours, centrifuged, washed twice with MB7H9, resuspended in RNA Later and stored at -80°C for RNA isolation. For treatment with Compound 2

(C2), Mtb H37Rv or its variants were grown in MB7H9 till O.D.$_{600}$ reached 0.2-0.3 and C2 was added at specified concentrations followed by removal of aliquots at the specified time points.

Homology model development and validation for MprA

The MprA protein sequence was collected from the Swiss-Prot Protein Databases (accession number: P9WGM9). A similarity search for MprA in the Protein Data Bank was performed using the Blast server. The protein similarity search identified similar protein structures belonging to the OmpR family in *M. tuberculosis* (PDB Ids: 3ROJ, 1YS7, 1YS6, 1KGS, 3F6P, 2ZWM and 1NXO) which had more than 48% sequence identity with MprA. These structures were used as templates to generate a consensus model. The modeled protein was energy minimized using the OPLS-2005 force-field. Both domains were composed of α-helices and β-strands (Jacobson *et al.*, 2004). Validation of the model obtained from Prime was performed by inspecting the psi/phi Ramachandran plot obtained from PROCHECK analysis (Laskowski *et al.*, 1993). The Ramachandran plot showed 82.8% of the residues in the most favorable region, 13.3% of the residues in the additionally allowed region, 1.5% of the residues in generously allowed region and 2.5% in the disallowed region. Thus, the majority of the amino acids were in a phi-psi distribution that is consistent with a right-handed α-helix and the model was reliable and of good quality. The PROVE test was applied to the final model to calculate the volumes of atoms in macromolecules using an algorithm which treats the atoms like hard spheres and calculates a statistical Z-score deviation for the model from highly resolved (2.0 Å or better) and refined (R-factor of 0.2 or better) PDB deposited structures (Pontius *et al.*, 1996). The G-factors, indicating the quality of the covalent, dihedral and overall bond angles, were 0.8° for H bond, 8.3° for omega angle and -0.3° for overall.

Docking studies

The crystal structures of Mtb response regulator proteins MtrA and RegX3 (PDB codes: 2GWR and 2OQR respectively) (Freidland *et al.*, 2007; King-Scott *et al.*, 2007) were retrieved from the protein data bank (PDB) and the protein structure models generated were utilized for structure-based drug design. Using protein preparation wizard of the Maestro software package (Sastry *et al.*, 2013) bond orders were assigned, missing hydrogens were added to heavy atoms and coordinates were predicted for the missing loops. Hydrogen-bonding network was optimized by reorienting hydroxyl and thiol groups, amide groups of asparagine and glutamine and the imidazole ring in histidine.

Protonation states of histidine, aspartic acid and glutamic acid, and tautomeric states of histidine were predicted at neutral pH. Finally, the resulting structure was energy minimized using with OPLS 2005 forcefield with a maximum RMSD of 0.30 Å for heavy atoms available in Schrodinger suite version 9.3. Sitemap identifies potential binding sites by linking together site-points that are most likely to contribute to protein-ligand or protein-protein binding (Halgren, 2009). The whole protein was used to analyze the binding site. Top 4 sites were generated based on the volume, D score (druggability score) and site score. Based on the scores and the area that it occupies near the amino acids L201, R202 and K204 (L202, R203, K205 for MprA) we selected one site and generated a grid box for docking of molecules. Grid was generated using Receptor grid generation in Glide module (Friesner *et al.*, 2006) (Schrodinger, LLC), default parameters were used and no constraints were applied. In another approach, a grid was generated by taking the centroid of aminoacids L201, R202 and K204 (L202, R203, K205 for MprA) for locating a grid, default parameters were used and no constraints were applied. The BITS database containing 2500 compounds was used in this study. Database molecules were prepared using Ligprep and Epik (Greenwood *et al.*, 2010) to expand protonation and tautomeric states at pH 7.0. Conformation sampling was performed for all molecules using the ConfGen (Watts *et al.*, 2010) search algorithm. We employed ConfGen with the OPLS-2005 forcefield and a duplicate pose elimination criterion of 1.0 Å RMSD to remove redundant conformers. A distance-dependent dielectric solvation treatment was used to screen electrostatic interactions. A maximum relative energy difference 10.0 kcal/mol was chosen to exclude high energy structures. All prepared database molecules were docked into two grids of the protein utilizing high throughput virtual screening (HTVS) scoring function to estimate the protein-ligand binding affinities. Ligands filtered from the HTVS were subjected to Glide SP (standard precision) docking. Post docking minimization was implemented to optimize the ligand geometries. Compounds with the best docking and glide scores were then subjected to the Glide XP (extra precision) docking. Final short listing of possible hit compounds was based on the visual inspection of the important amino acids in the active site cleft involved in binding and the hydrophobic interactions.

Synthesis of compounds 2 and 6

Compound 2 (C2) Synthesis: Briefly, 2-Imino-3-(3-(trifluoromethyl)phenyl)thiazolidin-4-one (0.25 g, 0.96 mmol), NaOAc (0.15 g, 1.92 mmoles) and 2-hydroxybenzaldehyde (0.14 g, 1.15 mmol) were

heated at 100 °C for 3 h in 2 ml acetic acid . Reaction mixture was filtered and washed with water, a small volume of ethanol and hexanes to afford title compound (0.38 g, 90%) as an off-white solid.

5-(2-Hydroxybenzylidene)-2-imino-3-(5-nitrothiazol-2-yl) thiazolidin-4-one: Color: White. Yield: 88%. 1H NMR (400 MHz, DMSO-d6, δ, ppm): 12.42 (s, 1H, OH), 9.70 (s, 1H, NH), 8.31 (s, 1H, Ar-H), 7.92 (d, J = 7.2 Hz, 1H, Ar-H), 7.81 (s, 1H, CH), 7.72-7.69 (m, 2H, Ar-H), 7.63 (t, J = 7.6 Hz, 1H, Ar-H). 13C NMR (100 MHz, DMSO-d6, δ, ppm): 172.6, 163.4, 156.4, 149.4, 137.4, 131.4, 128.5, 126.4, 125.2, 123.8, 121.4, 120.4, 119.3. MS (EI,m/z (%)): 349 [M+H]+. Anal. calcd. for C13H8N4O4S2: C, 44.82; H, 2.31; N, 16.08. Found: C, 44.92; H, 2.41; N, 16.12%.

Compound 6 (C6) Synthesis: Synthesis was done as reported by Poyraz *et al.* (2013). Briefly, to a well-stirred solution of 3-((3-alkyl-4-oxothiazolidin-2-ylidene) amino) benzoic acid (1mmol) in ethanol (6 ml) was added dropwise piperidine (0.3mmol) and the corresponding aldehyde (1.1 mmol). The reaction mixture was refluxed for 6 h, monitored for completion (by TLC and LC-MS) and evaporated to dryness. The residue was diluted with 10 ml water and acidified with 2 N HCl to pH 2. Dichloromethane (1 × 20 mL) was added to the reaction mixture, and the layers were separated. The aqueous layer was again extracted with 15 ml dichloromethane. The combined organic layer was washed with 10 ml water and 10 ml brine, dried over anhydrous sodium sulfate, and evaporated to dryness. The residue was then purified by column chromatography using hexane:ethyl acetate as eluent to give the desired product in good yield as described below.

3-((Z)-((Z)-5-(4-(carboxymethoxy) benzylidene)-3-allyl-4-oxothiazolidin-2- ylidene) amino) benzoic acid. Buff coloured solid; Yield (63%); 1 H NMR (DMSO-d6): δH. 4.52 (d, 2H, J = 5.3Hz), 4.63 (s, 2H), 5.12-5.21 (m, 2H), 5.74-5.89 (m, 1H), 6.99-7.28 (m, 3H), 7.65-8.09 (m, 6H). 13C NMR (DMSO-d6): δc. 170.4, 166.5, 163.4, 160.3, 156.8, 148.6, 141.5, 132.1, 131.1, 130, 129.7, 127, 126.8, 125.8, 122.8, 117.1, 115.8, 113.7, 63.9, 42.3. EIMS m/z (Calcd.for C22H18N2O6S:438.45); Found:437.04 (M-H)- . Anal Calcd for C22H18N2O6S: C, 60.27; H, 4.14; N, 6.39. Found: C, 60.28; H, 4.09; N, 6.41.

Alamar Blue Assay

Bacterial suspensions (100 µl) were added to 96-well plates in the absence or presence of compound (2 or 6) and incubated at 37^0C for at least 4 days. Alamar Blue solution (20 µl) was added and incubated for 24 h. The change in color from pink to blue was measured.

Evaluation of bacterial replication using the plasmid pBP10

Mtb H37Rv competent cells were electroporated with pBP10 plasmid (a kind gift from Dr. David Sherman, University of Washington, Seattle, USA) and colonies were selected on kanamycin plates. A single colony was grown to an O.D. $_{600}$ of 1, and cells were stored in 15% glycerol at -80°C for further use. A log phase culture of Mtb-pBP10 in 7H9 and 10% ADC was maintained with or without C 2 in the absence of kanamycin for ~20 generations, sub-culturing every 3-4 days. The culture was plated on 7H10 with or without kanamycin (30µg/ml) for CFU measurements and the loss of plasmid was quantified as the CFU on kanamycin plates divided by the CFU on plates without kanamycin.

Culture filtrate preparation and Western blotting

Mtb Erdman culture filtrates were prepared by growing in MB 7H9 till an O.D.$_{600}$ of 0.3-0.4. The bacteria were washed and reconstituted in Sauton's media without Tween 80 to O.D. 0.1 followed by incubation at 37 °C with shaking at 50 rpm. Aliquots were collected on day 7 and centrifuged to remove cells. The supernatants were filtered and then concentrated using a Vivaspin 3 kDa MWCO. For preparation of lysates, cells were disrupted in a bead beater. Proteins were extracted and immunoblotted with ESAT-6 or GroEL antibody.

Cell culture and infection

The murine macrophage RAW264.7 cell line was obtained from the National Centre for Cell Science, Pune and cultured in DMEM supplemented with 10% fetal bovine serum, 4mM glutamine and penicillin/streptomycin at 37^0 C in a humidified atmosphere at 5% CO_2. Human monocytes were obtained from blood from healthy donors by density gradient centrifugation (Welin *et al*., 2008). Monocytes were cultured in media containing 0.2ng/ml human macrophage colony-stimulating factor (M-CSF) for 7 days to allow differentiation. Bone marrow was flushed out from the bones of balb/c mice and monocytes were cultured in 20 ng/ml of murine M-CSF for 7-8 days to allow them to mature (Boone *et al*., 2004). For infections, bacteria were grown in MB 7H9 broth (Difco) supplemented with 0.05% Tween 80 and 10% ADC up to OD_{600} of ≤ 0.2. Bacterial clumps were removed by passing through a 27 gauge syringe thrice. Infection was carried out for 4 h followed by washing and incubation in gentamycin containing medium for 2 h. The cells were further incubated in DMEM supplemented with 10% FBS for different time points as required.

Microscopy

RAW 264.7 cells or hMDMs or BMDMs were seeded at ~$3x10^5$ cells per well on 18 mm cover slips. After infection and treatment with C2 or isoniazid (INH), the cells were fixed with 4% paraformaldehyde for 10 min at 25°C followed by permeabilisation with 0.01% Triton X-100 in PBS for 10 min at 25°C. The cells were then treated with 10% BSA in PBS for 90 min at 25°C followed by treatment with anti-LC3 antibody at 4°C overnight. Cells were then washed with PBS and treated with Alexa488 conjugated secondary antibody for 90 min at 25°C. Cells were visualized in a Leica confocal microscope.

Bacterial CFU determinations

RAW264.7 cells or hMDMs or BMDMs were plated in 48 well plates. Cells were infected with Mtb and treated with C2 or C6 for the desired periods of time. Infected cells were lysed in distilled water with 0.06% SDS. Serial dilutions of homogenates were plated on 7H11 agar plates supplemented with 10% OADC and incubated at 37^0C for 3–4 weeks. CFUs were calculated in triplicate using standard procedures. For Alamar Blue assays, bacteria from the cell lysates were washed and transferred to a 96 well plate in MB 7H9. Alamar Blue was added and readings were taken upon development of color.

Nitric oxide (NO) assay

For NO (nitrite) measurements, RAW264.7 or MDMs or BMDMs were infected with Mtb H37Rv. Control and infected supernatants obtained in the absence or presence of C2, were used for the measurement of nitrite using Griess reagent (Invitrogen), according to the manufacturer's instructions. Supernatant nitrite concentrations were measured by reading absorbance at 540 nm and calculated using a standard curve.

List of oligonucleotides used in this study

Primers used for cloning, site-directed mutagenesis (SDM), knockout (KO) generation, complementation (CT), and oligonucleotides used in EMSA and for SPR

Primer list

Serial No.	Primer name	Primer sequence	Purpose
P1	RegX3Mtb F	5’AAGGATCCATATGACCAGTGTGTTGA3’	Cloning
P2	RegX3Mtb R	5’AGAATTCCTAGCCCTCGAGTTTGTAG3’	Cloning
P3	SenX3Mtb F	5’ATAGGATCCCGGCTGACGTCGCG3’	Cloning
P4	SenX3Mtb R	5’AAAAAGCTTTCATCGGCTCAGCTCTTC3’	Cloning
P5	MprAMtb F	5’ATAGGATCCGGTGCGAATTCTTGTC3’	Cloning
P6	MprAMtb R	5’ATGGTACCTCAGGGTGGTGTTTCAC3’	Cloning
P7	MprBMtb F	5’ATAGGATCCTACAGCGACATCGACAACCA3’	Cloning
P8	MprBMtb R	5’AAAAAGCTTCTAGGTTGCGCGCG3’	Cloning
P9	MtrAMtb F	5’AGGTACCCATATGGACACCATGAGGCA3’	Cloning
P10	MtrAMtb R	5’AAAAAGCTTCGGAGGTCCGGCCTTG3’	Cloning
P11	regX3L201A F	5’CATGTCAAGCGGGCACGCTCCAAGAT 3’	SDM
P12	regX3L201A R	5’ATCTTGGAGCGTGCCCGCTTGACATG 3’	SDM
P13	regX3R202A F	5’CAAGCGGCTGGCCTCCAAGATCG 3’	SDM
P14	regX3R202A R	5’CGATCTTGGAGGCCAGCCGCTTG 3’	SDM
P15	regX3S203A F	5’CGGCTGCGCGCCAAGATCGAA 3’	SDM
P16	regX3S203A R	5’TTCGATCTTGGCGCGCAGCCG3’	SDM
P17	regX3K204A F	5’CTGCGCTCCGCCATCGAAGCC 3’	SDM
P18	regX3K204AR	5’GGCTTCGATGGCGGAGCGCAG 3’	SDM
P19	mtrAL201A F	5’GTGCATGTCCAGCGTGCCCGGGCCAAGGTCGA	SDM

		A3’	
P20	mtrAL201A R	5’TTCGACCTTGGCCCGGGCACGCTGGACATGCA C3’	SDM
P21	mtrAR202A F	5’CATGTCCAGCGTCTGGCCGCCAAGGTCGAAAA G3’	SDM
P22	mtrAR202A R	5’CTTTTCGACCTTGGCGGCCAGACGCTGGACATG 3’	SDM
P23	mtrAK204A F	5’CAGCGTCTGCGGGCCGCCGTCGAAAAGGATCC C3’	SDM
P24	mtrAK204A R	5’GGGATCCTTTTCGACGGCGGCCCGCAGACGCT G3’	SDM
P25	mprA R202A F	5’ACGTCGGGTATCTAGCACGCAAGACCGAGGC 3’	SDM
P26	mprA R202A R	5’GCCTCGGTCTTGCGTGCTAGATACCCGACGT 3’	SDM
P27	mprA L201A F	5’TCTACGTCGGGTATGCACGCCGCAAGACCGA 3’	SDM
P28	mprA L201A R	5’TCGGTCTTGCGGCGTGCATACCCGACGTAGA 3’	SDM
P29	mprA R203A F	5’TCGGGTATCTACGCGCAAAGACCGAGGCCGA 3’	SDM
P30	mprA R203A R	5’TCGGCCTCGGTCTTTGCGCGTAGATACCCGA 3’	SDM
P31	mprA K204A F	5’GGTATCTACGCCGCGCAACCGAGGCCGACGG 3’	SDM
P32	mprA K20A R	5’CCGTCGGCCTCGGTTGCGCGGCGTAGATACC 3’	SDM
P33	mprAupcy5 F	5’CCAATGTGGCTGATGTGGCTAA3’	EMSA/SPR
P34	mprAup R	5’CGTCAACGACAAGAATTCGCA3’	EMSA/SPR
P35	ppK1upcy5 F	5’ACCGGCTTGGGACCGGCA 3’	EMSA/SPR
P36	ppK1up R	5’GCGCCCGAACGGCGGTGC3’	EMSA/SPR
P37	fbpBupcy5 F	5’GCGGTAACCGATACGGAAATGAGACGACTTTGC GCCCGAATCGACATTTGGCCTCC3’	EMSA/SPR
P38	fbpBup R	5’CCAAGCTCGAATCTTTCGGCTCACG3’	EMSA/SPR

P39	mprAUEcoRvF	5’AGATATCCGTCGCCGATCAATGGG3’	KO
P40	mprAUXbaR	5’CGTCTAGATCAACGACAAGAATTCGCACG3’	KO
P41	mprADHindF	5’ATAAAGCTTGAAACACCACCCTGATG3’	KO
P42	mprADSpeR	5’ATAACTAGTGTACGCAATTCATGTCCG3’	KO
P43	regX3UEcoRV	5’TGATATCTCGATACGATTGTGTCGGAAG3’	KO
P44	regX3UXba1	5’CGTCTAGACACACTGGTCATCAGCG3’	KO
P45	regX3DNco1	5’ATACCATGGAAACTCGAGGGCTAGC3’	KO
P46	rgX3DSpe1	5’ATAACTAGTCTGGCCGACGCGGTC3’	KO
P47	mprABpmv306F	5’ ATAAAGCTTGACATCGAAATCCTCCTGAC3’	CT
P48	mprABpmv306R	5’GCTCTAGAAACAGTAACTGCACAACTAGGTT3’	CT
P49	RegX3pmv306R	5’ATAAAGCTTAACCCGCAGCTGTCCAT3’	CT
P50	RegX3pmv306F	5’GCTCTAGAGCTACGACCTGGATTCCG3’	CT
P51 (Q1)	RegX3U F	5’ATGGATCCGAGCGTAGCGATGAGGTG3’	KO verification
P52 (Q2)	RegX3D R	5’AAAAAGCTTCCATCCGACGGGCACC3’	KO verification
P53 (A1)	mprA200bp F	5’ATAACTAGTCATTGGTGGAACGTGGTGC3’	KO verification
P54 (A2)	mprA200bp R	5’ATAAAGCTTGTACCCTCGATTGCCTTACC3’	KO verification
P55	hspX F	5’TCTGAACGGCGGTTGGCAGACAAC3’	EMSA
P56	hspX R	5’CGGGAAGGGTGGTGGCCATTTG3’	EMSA

Primers used for qRT-PCR and semi quantitative RT-PCR

Serial No.	Primer Name	Primer Sequence 5'-3'
R1	gltA1RT F	ATTCGTTGACCTACCGGGGATA
R2	gltA1RT R	CAGCATCGAGCGGTCCAC
R3	pstS3sRT F	ATTTGGTGCCGCCGTAGGT
R4	pstS3sRT R	CGACCCACTGGCTTTGAGTG
R5	sigERT F	TGTTCCGGTCGGTCCAGA
R6	sigERT R	GGTGATGCGGTGTAGCCAG
R7	sigBRT F	GCGCTTCGGCCTGGA
R8	sigBRT R	GCCCGAATAGTTTGCCGATT
R9	mprART F	CGCTTTCCTTCAATGGCTATTC
R10	mprART R	TCATGTCGAGCGCCTCAA
R11	mtrART F	TCATCGGCGACGGTACTCAG
R12	mtrART R	GGGCAGCATCAAATCCAATAACAC
R13	regX3RT F	CAGCGTTCCGGTGATCATG
R14	regX3RT R	CAGGCCGACCACCTTGTC
R15	espART F	CAACCAAGGGGGTATCCTTT
R16	espART R	CTGATGAGCTGACGATCGAG
R17	espRRT F	AACCGCCTGTTCGACACGGTTTAT
R18	espRRT R	TTCCTGAGCGTAGCTGTGATAGGT
R19	esxART F	GTACCAGGGTGTCCAGCAAAA
R20	esxART R	GCAGCGCGTTGTTCAGC
R21	rpfBRT F	ATGTTGCGCCTGGTAGTCG
R22	rpfBRT R	CGTCGACTGAGAACCCGT

R23	dnaNRT F	GTTGGACGAGCCGAGGAAGA
R24	dnaNRT R	GGACCGTTGCCATTCAGACC
R25	ftsIsRT F	TGGTGCAACGCGATCCGATG
R26	ftsIsRT R	CAGCCAGGGTTGATCTGCTG
R27	Rv0081RT F	GTCGGTGAGTTGCTGTCCT
R28	Rv0081RT R	GCAATCGAATAGATCATCGC
R29	cydART F	CGTGTCATCGAAGTGCCCTATGTG
R30	cydART R	AGGAATACCAGCGCTTCGG

Supplemental references.

Boone, D.L. *et al.* The ubiquitin-modifying enzyme A20 is required for termination of Toll-like receptor responses. *Nat. Immunol*. **5**, 1052–1060 (2004).

Friesner, R.A. *et al*. Extra precision glide: docking and scoring incorporating a model of hydrophobic enclosure for protein-ligand complexes. *J. Med. Chem*. **49**, 6177–6196 (2006)..

Greenwood, J.R., Calkins, D., Sullivan, A.P. & Shelley, J.C. Towards the comprehensive, rapid, and accurate prediction of the favorable tautomeric states of drug-like molecules in aqueous solution. *J. Comput. Aided Mol. Des*. **24**, 591-604 (2010).

Halgren, T. Identifying and characterizing binding sites and assessing druggability. *J. Chem. Inf. Model.* **49**, 377–389 (2009).

Jacobson, M.P. *et al*. Hierarchical approach to all-atom protein loop prediction*. Proteins: Structure, Function and Bioinformatics* **55**, 351-367 (2004).

Laskowski, R.A., MacArthur, M.W., Moss, D.S. & Thornton, J.M. PROCHECK - a program to check the stereochemical quality of protein structures. *J. App. Cryst*. **26**, 283-291 (1993).

Pontius, J., Richelle, J. & Wodak, S.J. Deviations from standard atomic volumes as a quality measure for protein crystal structures. *J. Mol. Biol*. **22**, 121-136. (1996).

Poyraz, O. *et al*. Structure guided design of novel thiazolidine inhibitors of O-acetyl serine sulfhydrylase from *Mycobacterium tuberculosis*. *J. Med. Chem*. **56**, 6457-6466 (2013).

Sastry, G.M., Adzhigirey, M., Day, T., Annabhimoju, R. & Sherman, W. Protein and ligand preparation: Parameters, protocols, and influence on virtual screening enrichments. *J. Comput. Aid. Mol. Des.* **27**, 221-234 (2013).

Vashisht, A., Prithvi Raj, D., Gupta, U.D., Bhat, R., & Tyagi, J.S. The α10 helix of DevR, the *Mycobacterium tuberculosis* dormancy response regulator, regulates its DNA binding and activity. *The FEBS Journal*. doi:10.1111/febs.13664 (2016).

Watts, K.S. *et al*. ConfGen: A conformational search method for efficient generation of bioactive conformers. *J.Chem. Inf. Model.* **50**, 534-546 (2010).

Welin, A. *et al*. Incorporation of *Mycobacterium tuberculosis* lipoarabinomannan into macrophage membrane rafts is a prerequisite for the phagosomal maturation block. *Infect Immun*. **76**, 2882–2887 (2008).
